# Synergistic Screening of Peptide-Based Biotechnological Drug Candidates for Neurodegenerative Diseases Using Yeast Display and Phage Display

**DOI:** 10.1021/acschemneuro.3c00248

**Published:** 2023-08-28

**Authors:** Cemile Elif Özçelik, Özge Beğli, Ahmet Hınçer, Recep Erdem Ahan, Mehmet Seçkin Kesici, Oğuzhan Oğuz, Talip Serkan Kasırga, Salih Özçubukçu, Urartu Özgür Şafak Şeker

**Affiliations:** † UNAM − Institute of Materials Science and Nanotechnology, Bilkent University, Ankara 06800, Turkey; ‡ Department of Chemistry, Faculty of Science, Middle East Technical University, Ankara 06800, Turkey; § Interdisciplinary Program in Neuroscience, Bilkent University, Ankara 06800, Turkey

**Keywords:** yeast surface display, phage display library, neurodegenerative disease, peptide-based drug discovery

## Abstract

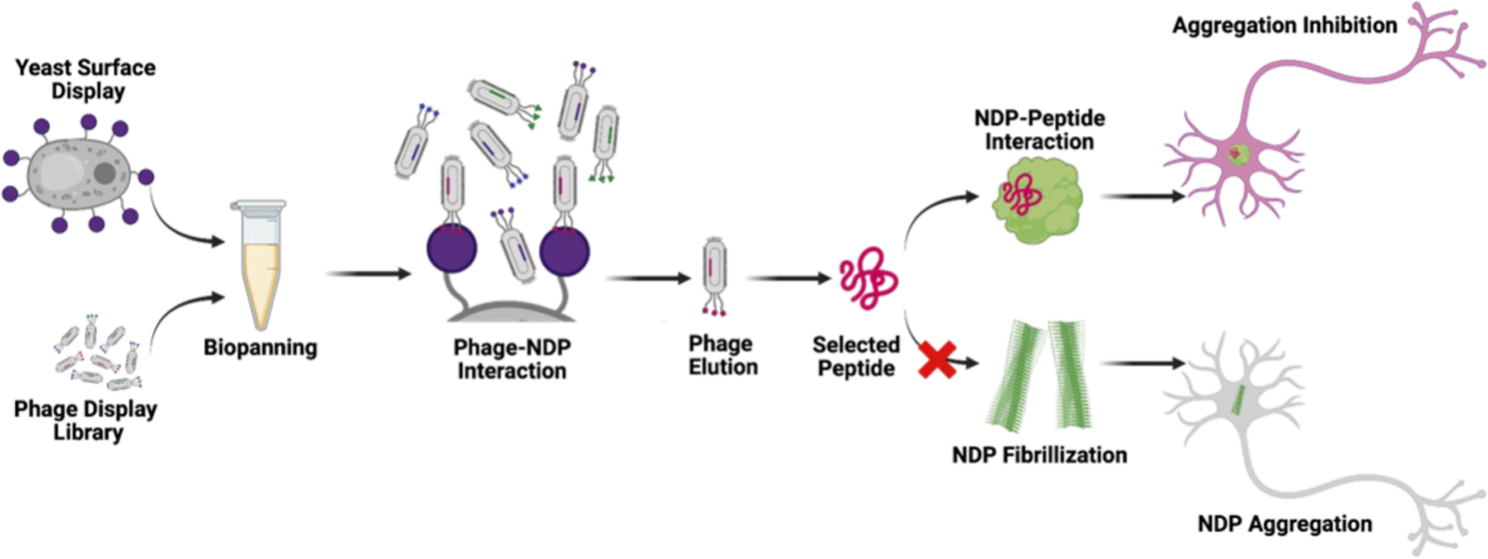

Peptide therapeutics are robust and promising molecules for treating diverse disease conditions. These molecules can be developed from naturally occurring or mimicking native peptides, through rational design and peptide libraries. We developed a new platform for the rapid screening of the peptide therapeutics for disease targets. In the course of the study, we aimed to employ our platform to screen a new generation of peptide therapeutic candidates against aggregation-prone protein targets. Two peptide drug candidates were screened for protein aggregation-prone diseases, namely, Parkinson’s and Alzheimer’s diseases. Currently, there are several therapeutic applications that are only effective in masking or slowing down symptom development. Nonetheless, different approaches are being developed for inhibiting amyloid aggregation in the secondary nucleation phase, which is critical for amyloid fibril formation. Instead of targeting secondary nucleated protein structures, we tried to inhibit the aggregation of monomeric amyloid units as a novel approach for halting the disease condition. To achieve this, we combined yeast surface display and phage display library platforms. We expressed α-synuclein, amyloid β_40_, and amyloid β_42_ on the yeast surface, and we selected peptides by using phage display library. After iterative biopanning cycles optimized for yeast cells, several peptides were selected for interaction studies. All of the peptides have been used for *in vitro* characterization methods, which are quartz crystal microbalance-dissipation (QCM-D) measurement, atomic force microscopy (AFM) imaging, dot-blotting, and ThT assay, and some of them have yielded promising results in blocking fibrillization. The rest of the peptides, although, interacted with amyloid units which made them usable as a sensor molecule candidate. Therefore, peptides selected by yeast surface display and phage display library combination are good choice for diverse disease-prone molecule inhibition, particularly those inhibiting fibrillization. Additionally, these selected peptides can be used as drugs and sensors to detect diseases quickly and halt disease progression.

## Introduction

Selection of synthetic peptide-based drug molecules started back in the 1980s with the first combinatorial library development, in which foreign DNA pieces were inserted in the phage genome to express random peptide regions in the coat protein of the phage.^1^ This approach has provided a tremendous development in the discovery of therapeutic peptides as accumulation of know-how of screening methodologies.^2^ With the emergence of potential therapeutic peptides, this journey culminated with the approval of more than 80 peptide-based drugs, as well as 400–600 peptides undergoing preclinical trials.^3,4^ In light of such peptide-based drug discoveries, display technologies have become the leading technology of peptide selection and screening for pharmaceutical applications.

Using display applications and combinatorial peptide library production, a large and diverse number of peptide pool screenings can be achieved against specific targets, from inorganic compounds to natural products.^5−7^ By taking advantage of this and the ease of displaying up to 10^10^ peptide sequences on the coat of the phage, phage display libraries have a wide range of applications, such as the discovery of peptides targeting specific tissues, organs, or tumors; molecular imaging; diagnosis and therapies for neurological disorders; epitope mapping; and novel antibody discoveries.^8−15^ The versatility of peptide screening provides a significant opportunity to select high-affinity bioactive peptides against specific targets.^16^ However, for pharmaceutical usage purposes, phage display library-derived peptides need more modifications to delivery, resistance to protease attacks, stability, specificity, and clearance as drugs from circulation.^17^ Still, phage display is one of the strongest strategies for peptide drug discovery, which was also appreciated with the one half of the 2018 Nobel Prize in Chemistry “for the phage display of peptides and antibodies” for jumping into a new era for drug molecule discovery by screening combinatorial phage display library.^18^

Alongside the phage display, yeast surface display is an impressive strategy to display a variety of proteins, especially mammalian proteins such as cytokines, surface proteins, antibodies, etc., that undergo post-translational modifications like disulfide bond formation for proper folding and activity, glycosylation as well as presenting the ease of culturing, immobilization, and manipulations.^19^ Displaying proteins in the yeast surface with eukaryotic processing after expression can increase the stability and decrease the vulnerability against temperature, pH, protease attacks, and diverse solvents.^20^ By taking advantage of yeast surface display technology as a eukaryotic expression system, many peptides and proteins can be expressed with high stability for protein engineering, and antibody and nanobody screening, as well as fully functionalized enzyme selection.^21−25^ Besides, the surface expression of intracellularly toxic proteins is a better approach for interaction studies. Especially in the case of pathogenic amyloids, the monomeric expression outside the yeast cell provides both fully active and proper protein forms and decreases the toxic effect compared to the case of that residing in the cytosol.^26^ Yeast surface display provides immobilization of such dynamic and problematic pathogenic amyloid precursors to discover drug molecules. To date, there are various approaches for antibody or drug molecule discovery for such proteins by using yeast surface display as well as phage display separately.^27−29^ During the usage of display systems, proteins are immobilized onto solid surfaces like ELISA plates, polystyrene tubes, and magnetic beads, or the screening and panning method requires an additional reporter expression or antibody labeling for sorting the library particles that interact with the target protein.^30−35^

Here, we developed a platform of a combination of phage display and yeast surface display to discover ligand molecules targeting dynamic molecules like amyloid precursors. In our platform, the monomeric versions of α-synuclein, amyloid β_40_, and amyloid β_42_ were expressed on the *Saccharomyces cerevisiae* surface as a fusion with the mating factor protein Aga2p to express them in a proper folding with post-translational modifications. Second, a phage-displayed peptide library is used for the screening of the potential amyloid formation blocking peptides. We employed precursor proteins related to two different disease conditions. As a proof of concept, we targeted the amyloid formation blocking in Alzheimer’s disease peptides amyloid β_40_ and amyloid β_42_ and Parkinson’s disease-related α-synuclein protein. First, these proteins were separately displayed on the yeast surface as a fusion of Aga2p. Second, for each of the precursor protein cases, M13 phage display library expressing 10^9^ different peptide sequences was introduced to select aggregation-blocking peptides. After the iterative selection of interacting peptides was completed by the biopanning method, peptide sequences were obtained. The peptides were synthetically produced and used for further in vitro characterizations such as their amyloid formation blocking potential. We carried out the protein–peptide interaction analysis by using quartz crystal microbalance (QCM). To evaluate the effect of the selected peptides on aggregation, fibrillization assay was performed for each protein and peptides, and we observed the amyloid assembly and prevention activities of the inhibitory peptides on the formation of amyloids in real time with atomic force microscopy imaging. Finally, we tried to validate our results by applying Thioflavin-T staining and dot-blotting. In this study, we showed that the combination of yeast and phage display systems forms a new generation of peptide-based drug screening platform. The platform provides living yeast cells for protein target displays, and displayed peptide libraries can be employed sequentially to fast screening of potential peptide-based drug candidates.

## Results and Discussions

### Cell Surface Display of Monomeric Forms of Neurodegenerative Proteins

**Figure 1:**
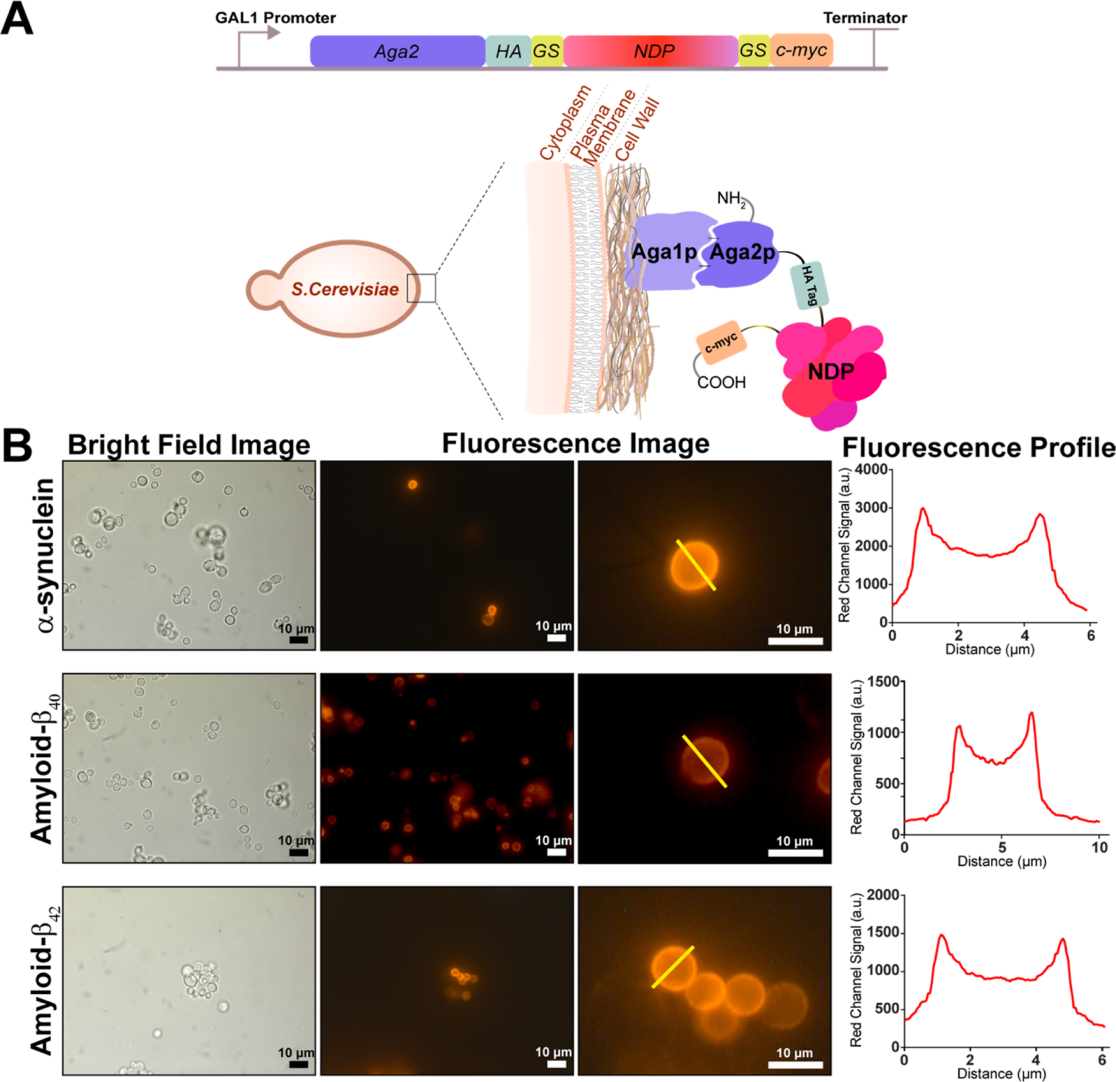
Yeast surface display system was utilized for screening NDPs as monomeric units to select interacting peptides for changing the fibrillization states. (A) Yeast surface display was achieved by fusing NDP genes to the *Aga2* gene under Gal1 promoter in EBY100 strain. The expression of NDP–Aga2p occurred after induction by galactose. (B) Induced yeast cells expressing NDPs were characterized by the ICC method. The immunostaining was applied without any membrane-solubilizing agents. Both bright-field and fluorescence images were taken to analyze the protein expression. The surface profiling was determined from one yeast cell by following the straight yellow line. The peaks of the fluorescence profile graphs are observed on the surface of the cells.

Regarding neurodegenerative amyloid formation, the monomeric versions of proteins are prone to produce primary nucleation, resulting in ordered amyloid aggregation.^36^ We foresee that inhibition of aggregation at this first step is achievable. To do so, we aimed to screen peptides that can strongly bind to monomeric units of the neurodegenerative proteins (NDPs). Then, we decided to continue to check the effect of peptides in the aggregation process as aggregation inhibitors. For this reason, we used yeast surface display expressing neurodegenerative proteins, which are α-synuclein, amyloid β_40_, and amyloid β_42_, as a fusion with Aga2p (Figures 1A and S1A). NDP–Aga2p was cloned individually into pETcon vector (Figure S1B–D, Addgene plasmid # 41522; http://n2t.net/addgene:41522; RRID:Addgene_41522). For full length expression and normalization of surface expression function, hemagglutinin (HA) tag and c-Myc tag were added at 3′ and 5′ ends of NDP genes, respectively. After cloning, sequences were verified by next-generation sequencing. Next, galactose induction was performed to express NDPs in *S. cerevisiae*, and expression characterization was analyzed by immunocytochemistry (ICC). c-Myc-tagged NDPs were labeled anti-Myc-tag antibody without any treatment of the cells with membrane-solubilizing agents. Fluorescence of the surface-displayed NDPs was achieved by detection of the Myc-tag antibody with its specific antibody conjugated with DyLight550 in order to indicate the surface display verification and displaying efficiency. Immunostained *S. cerevisiae* cells were examined by fluorescence microscopy to prove the surface display of the target proteins We observed that immunostained cells expressing amyloid β_40_–Aga2p fusion, amyloid β_42_–Aga2p fusion, and α-synuclein–Aga2p fusion proteins appeared on the cell surface as shown in Figure 1. Additionally, bright-field imaging data showed that there was not any high level of cell aggregation due to NDP interaction (Figure 1B). This is especially important showing that the target proteins, which are prone to aggregate formation in their purified forms, are being displayed in monomeric subunits. Also, fluorescence emission profiles of the cell membranes were obtained for each of the target proteins based on the immunostaining and intensities. We verified the display of the target proteins and provided the fluorescence line profiles in the presence of the target proteins.

According to the fluorescence emission profile data, each NDP-expressing cell showed homogenized and well-displayed protein expression on the surface, which is used as the verification of the cell surface display of proteins of interest. It is also evident from Figure 1B that the expressed proteins were located at the cell membrane. Although fluorescence emission profiles were similar throughout the cell membranes, the red fluorescence signal levels for single NDP-expressing cells were different. However, these differences did not affect the use of cell cultures as elements of target displaying. Furthermore, the variation in expression levels for each NDP in the yeast cell cultures allowed us to assess the efficiency of peptide selection and enrichment ratio of phage particles after each cycle of biopanning.

**Figure 2:**
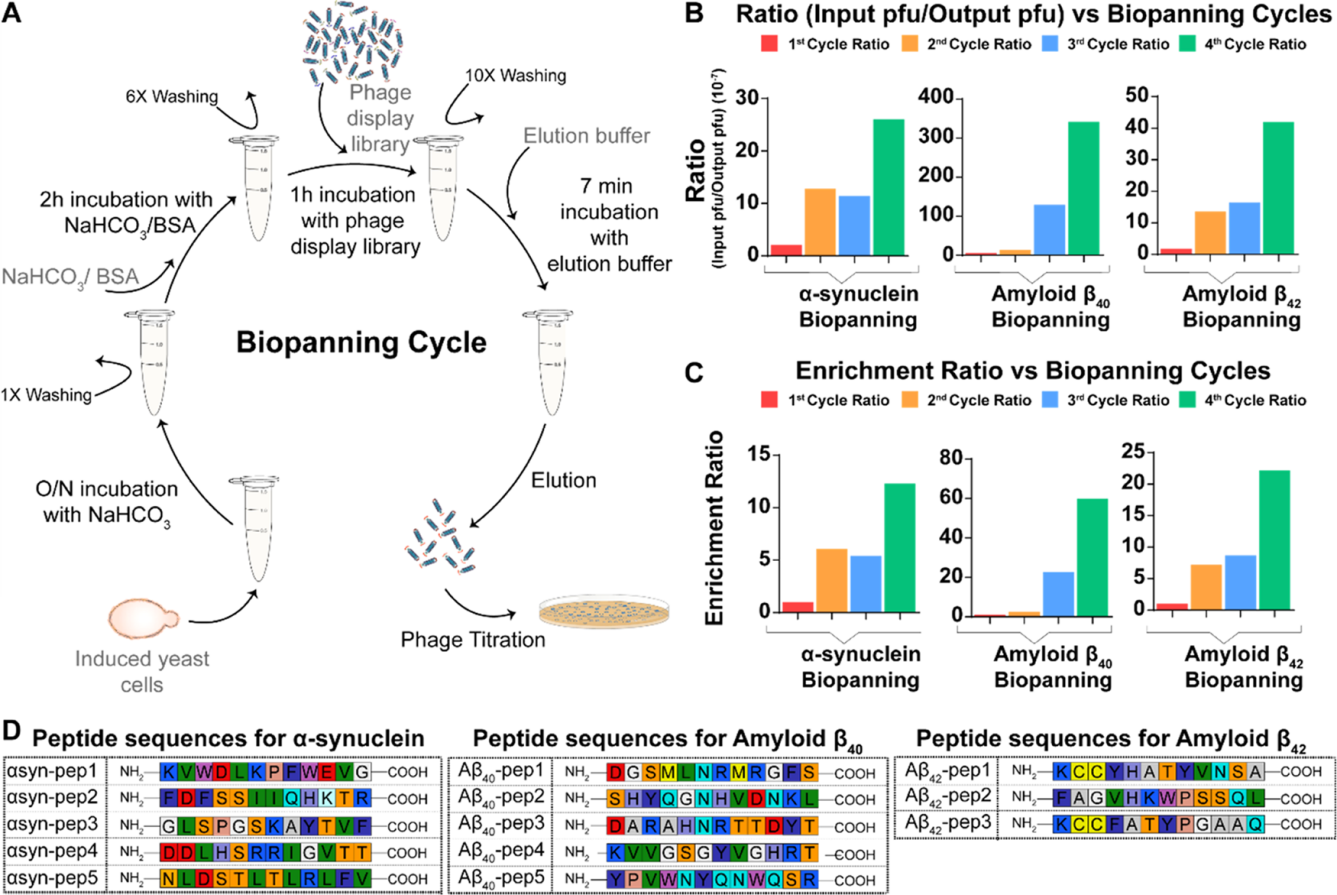
Peptide selection against NDPs was achieved by biopanning protocol with iterative cycles. (A) Yeast surface display and phage display library combination for peptide screening was achieved through optimized biopanning protocol by using yeast cells resuspended in appropriate buffers. There was no immobilization of cells to a solid surface throughout the protocol. (B) During each cycle, input phage forming units and interacting output phage forming units were recorded to decide the ratio for α-synuclein-, amyloid β_40_-, and amyloid β_42_-specific phage pool sizes. (C) Enrichment ratios for α-synuclein, amyloid β_40_, and amyloid β_42_ were calculated to show the increase in specificity of the obtained phage pool. (D) Selected peptides were sequenced for each NDP. Peptides were selected randomly from the sequenced phage particles. The amino acids were colored according to universal amino acid color codes, which showed their diversity. There were no continuous overlaps for no more than two amino acids.

Following the display of the target proteins, we exploited a commercial phage-displayed peptide library to select peptides that inhibit aggregation of neurodegenerative amyloids within this study. The commercial library size was around 10^9^ different peptides. After amplification of the library by following the manufacturer’s protocol, the common panning method for yeast cells was redesigned by using *S. cerevisiae* cells as liquid cultures instead of immobilization to any surfaces in order to prevent cell loss during iterative washing steps. In our approach, Eppendorf Protein LoBind microcentrifuge tubes (Eppendorf, Hauppauge, NY) were used during biopanning cycles to prevent unwanted adhesion of proteins and cells on tube walls. Biopanning protocol using a suspension yeast cell display system provided an opportunity to screen a vast number of cells in a relatively larger volume of target cell culture as well as simplicity in the application. Before selecting peptides, preselection was done against the complete empty *S. cerevisiae* cells not displaying any NDPs but the cell mating factors to prevent nonspecific interaction between phage particles and yeast surface-displayed elements other than NDPs. Unbound phage particles were eluted and amplified for further screening against NDP-displaying yeast cells. In the monomeric NDP-specific peptide selection step, four iterative cycles of biopanning against each NDP were performed separately (Figure 2A).

At the beginning of each cycle, the library size was fixed at about 10^9^ pfu as the input phage amount. During the iterative cycles, the input and output phage particles were recorded (Figure 2B). To show the increase in the specificity of phages obtained in the previous cycle, enrichment ratios were calculated by dividing the ratio result (output pfu/input pfu) by the first ratio result (Figure 2C). For each biopanning cycle, slight increases were observed in the interacted phage particle pool size for α-synuclein and amyloid β_42_ and a sharp increase for amyloid β_40_. These resulted in an increasing trend in enrichment ratios. These results were related to data obtained by ICC. Owing to the heterogeneity in the induced cell culture, the cultures having more immunostained cells presented a higher enrichment ratio value. Consequently, the increase in amyloid β_40_ enrichment was higher than amyloid β_42_ and α-synuclein enrichment ratios. After the last cycle of biopanning was achieved, phage particle elution was titered to obtain a single clone of each particle. The genomic regions of every phage particle having different peptide sequences in the *pIII* gene of the M13 phage encoding for pIII coat protein were sequenced by Sanger Sequencing (Genewiz, NJ). Following sequencing, five of the various peptide sequences for α-synuclein and amyloid β_40_, and three peptide sequences for amyloid β_42_ were chosen to characterize the effect of peptides in aggregation (Figure 2D). Peptides were synthesized by solid-state peptide synthesis (Supplementary Methods). Synthesized peptides were dissolved in 1× PBS for further use.

**Figure 3:**
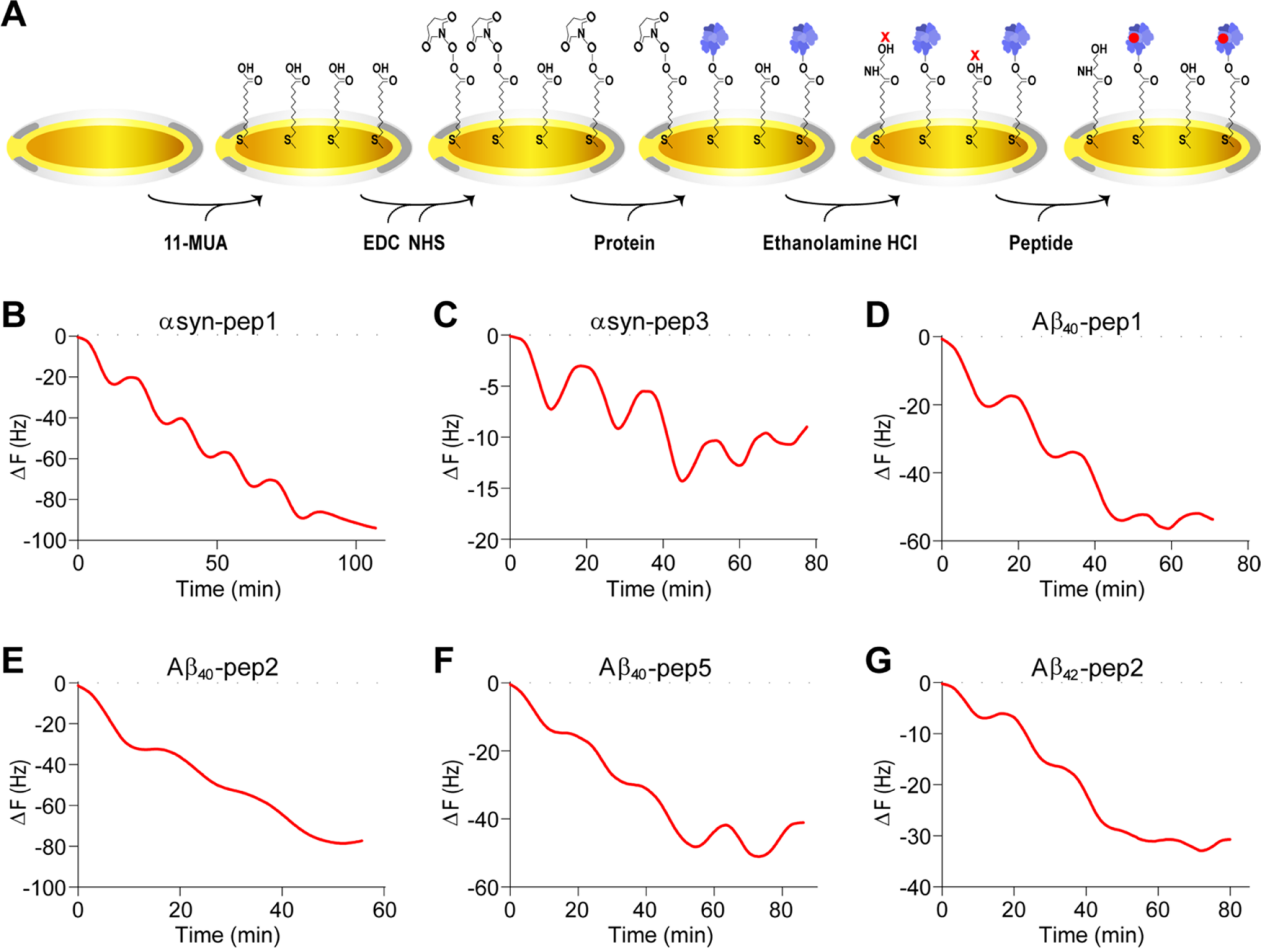
QCM analysis was applied to determine the protein–peptide interaction by evaluating frequency changes. (A) The chip preparation was achieved on a gold chip coated by 11-MUA. EDC/NHS surface activation was applied to immobilize the target protein. After NDP was attached to the activated surface, deactivation was done by 1 M ethanolamine HCl addition. The peptide was introduced to the protein-coated chip. For each peptide addition to the buffer flow in the chamber, Δ*F* decreased from 0 to more negative values. During wash steps, Δ*F* changed with less negative values, which creates an increase in the line and peak. Overall, Δ*F* became more negative at the end of the analysis. (B, C) Frequency changes for αsyn-pep1 and αsyn-pep3 with α-synuclein-coated chips were analyzed during each peptide addition and washing. (D–F) Aβ_40_-pep1, Aβ_40_-pep2, and Aβ_40_-pep5 were delivered onto amyloid β_40_-coated gold chips, individually. Frequency changes were recorded during each peptide addition and wash. (G) Aβ_42_-pep2 was analyzed by delivering onto the Aβ_42_-coated chip, and frequency changes were recorded. (For all NDP immobilization, Δ*F* results are shown in Figure S3).

After completion of the screening and selection of the peptides, we carried out further analysis of the inhibitory effects. Before characterizing the interaction of peptides with NDPs, monomeric amyloid β_40_ and amyloid β_40_ were used (GenScript), while α-synuclein was purified by the fast protein liquid chromatography (FPLC) method after expressed in *Escherichia coli* BL21 (DE3) (Figure S2). To increase the solubility, we expressed α-synuclein as a fusion of glutathione S-transferase (GST). After purification, we removed the GST tag by TEV protease and obtained monomeric α-synuclein protein for further use (Figure S2A–F). The first analysis relied on the protein–peptide interaction which was determined by using a mass-sensitive technique known as the quartz crystal microbalance device. The QCM measures the frequency change of the gold-coated quartz crystal sensor upon mass accumulation, providing a highly sensitive means of monitoring interactions.^37^ In this study, we monitored the interaction of inhibitory peptides with NDPs on the sensor’s surface. During QCM analysis, we recorded two frequency change measurements. The first one was done for the immobilization of NDP on the gold chip. First, the chip was coated with 11-mercaptoundecanoic acid (MUA) overnight, and then 1-ethyl-3-(3-dimethylaminopropyl)carbodiimide (EDC) was added to the QCM peristaltic pumping system. EDC created a decrease in frequency change. For coupling reaction, N-hydroxysuccinimide (NHS) was added to the chip surface. NHS created an increase in frequency change. After washing with 1× PBS, NDP was introduced to the chamber, and there was a decrease in frequency change. To remove the unbound proteins, the chip was washed. Deactivation of the chip was achieved by ethanolamine HCl addition to the chamber. Each change in the frequency represented a change in the mass dissipation on the chip. The second measurement was done for peptide addition to the QCM chamber. Peptide solution was added repeatedly with the same concentration, and between each peptide addition step, the chip was washed to remove unbound peptides. For each peptide addition to the flow, there was a decrease in frequency change because of the mass accumulation onto the chip. When the peptide interacted with NDP, there was a small increase in frequency change after wash steps. Overall, the decrease and increase in frequency change indicate that mass accumulation and dissipation on the gold chip occur, respectively (Figure 3A).

The binding analysis of α-synuclein peptides was conducted with five binding peptides. For each peptide, first monomeric α-synuclein was immobilized onto the gold chip. Changes in frequency during immobilization of α-synuclein were recorded before peptide analysis. Among them, αsyn-pep1 showed the most significant gradual drop in frequencies with a mass addition of 878 ng/cm^2^ onto the α-synuclein-coated chip with mass accumulation of 1778 ng·cm^–2^ (Figures 3B and S3A). Similarly, αsyn-pep3 interacted with a 536 ng·cm^–2^ α-synuclein-coated surface with a gradual decrease in frequencies and a mass accumulation of 136 ng/cm^2^ (Figures 3C and S3B). For the remaining αsyn-peptides, there was no clear and well-patterned gradual change in frequencies, although there were mass accumulations of 427, 27.2, and 3.8 ng/cm^2^ for αsyn-pep2, αsyn-pep4, and αsyn-pep5, respectively (Figure S4A–C).

We analyzed five different amyloid β_40_ peptides and found that each peptide had a unique interaction pattern with amyloid β_40_. Aβ_40_-pep1 showed a well-defined, gradual drop in frequency with each peptide addition, resulting in a mass accumulation of 704 ng/cm^2^ on the 4536 ng·cm^–2^ amyloid β_40_-coated surface (Figures 3B and S3C). For Aβ_40_-pep2, the frequency change decreased continuously between each peptide addition, without a sharp, gradual pattern, resulting in a mass accumulation of 786 ng/cm^2^ on the 5090 ng·cm^–2^ amyloid β_40_-coated surface (Figures 3C and S3D).

In the case of Aβ_40_-pep5 addition, there was a gradual drop in frequency for each peptide addition step, and the mass accumulation of Aβ_40_-pep5 was calculated as 715 ng/cm^2^ to the amyloid β_40_-coated surface with a mass accumulation of 4648 ng·cm^–2^ (Figures 3D and S3E). For Aβ_40_-pep3, there were gradual but small decreases in frequencies during each peptide addition step, with a mass accumulation of 405 ng/cm^2^ (Figure S4D). On the other hand, Aβ_40_-pep4 showed no interaction with the monomeric amyloid β_40_ protein since there was no frequency change between each peptide addition followed by a wash step. The small frequency change corresponded to 9.5 ng/cm^2^ mass accumulation, which indicates no significant and specific interaction between Aβ_40_-pep4 and amyloid β_40_ (Figure S4E).

When we evaluated the interaction of the three peptides with amyloid β_42_, Aβ_42_-pep2 showed a gradual decrease in frequencies between each peptide addition, with a mass accumulation of 442 ng/cm^2^ on the 1106 ng·cm^–2^ amyloid β_42_-coated chip (Figures 3E and S3F). Aβ_42_-pep1 showed a more well-defined gradual drop in frequencies with lesser peptide accumulation onto the amyloid β_42_ surface, with a value of 134 ng/cm^2^ (Figure S4F).

In contrast, there was no optimal pattern for frequency changes for Aβ_42_-pep3 during peptide addition and wash steps. Still, a mass accumulation was calculated as 100 ng/cm^2^, although the frequency change during peptide addition was not significant and apparent (Figure S4G).

As a negative control for evaluation of interaction between NDPs and the actual candidate peptides, we also used randomly synthesized peptide (KTWMDGFFSYGT) in QCM analysis (Figure S5). We checked only α-synuclein and amyloid β_42_ with the negative control peptide. In the case of α-synuclein, first we coated the gold chip with α-synuclein with a mass accumulation of 655 ng·cm^–2^. After the chip was coated, 1000 μM negative control peptide was added to the chamber repeatedly. Between each repeat, the chip was washed to remove unbound peptide molecules. At the end of the analysis, there was no significant interaction of negative control peptide with α-synuclein. Especially, frequency changes were relatively closer at the beginning of the peptide addition for the first time to the chamber and at the end of the first wash. When the analysis was completed, the peptide accumulation mass was 12.4 ng·cm^–2^ (Figure S5A). For the interaction between amyloid β_42_ and negative control peptide, amyloid β_42_ was coated onto the gold chip surface with 955.8 ng·cm^–2^ mass accumulation. Negative control peptide accumulation was 7.05 ng·cm^–2^. The decrease in frequency change during peptide addition and increase in frequency change after wash steps indicated that there was no specific peptide interaction with amyloid β_42_ (Figure S5B).

Thus, peptides that interacted with NDPs created a considerable mass accumulation on NDP-coated surfaces. The mass accumulation caused a decrease in frequency change during peptide addition in the system flow. Additionally, we demonstrated that the strong interaction between peptides and NDPs exhibited clear gradual changes in frequencies.

### Imaging of the Inhibitory Effect of Peptides on Amyloid Formation

**Figure 4:**
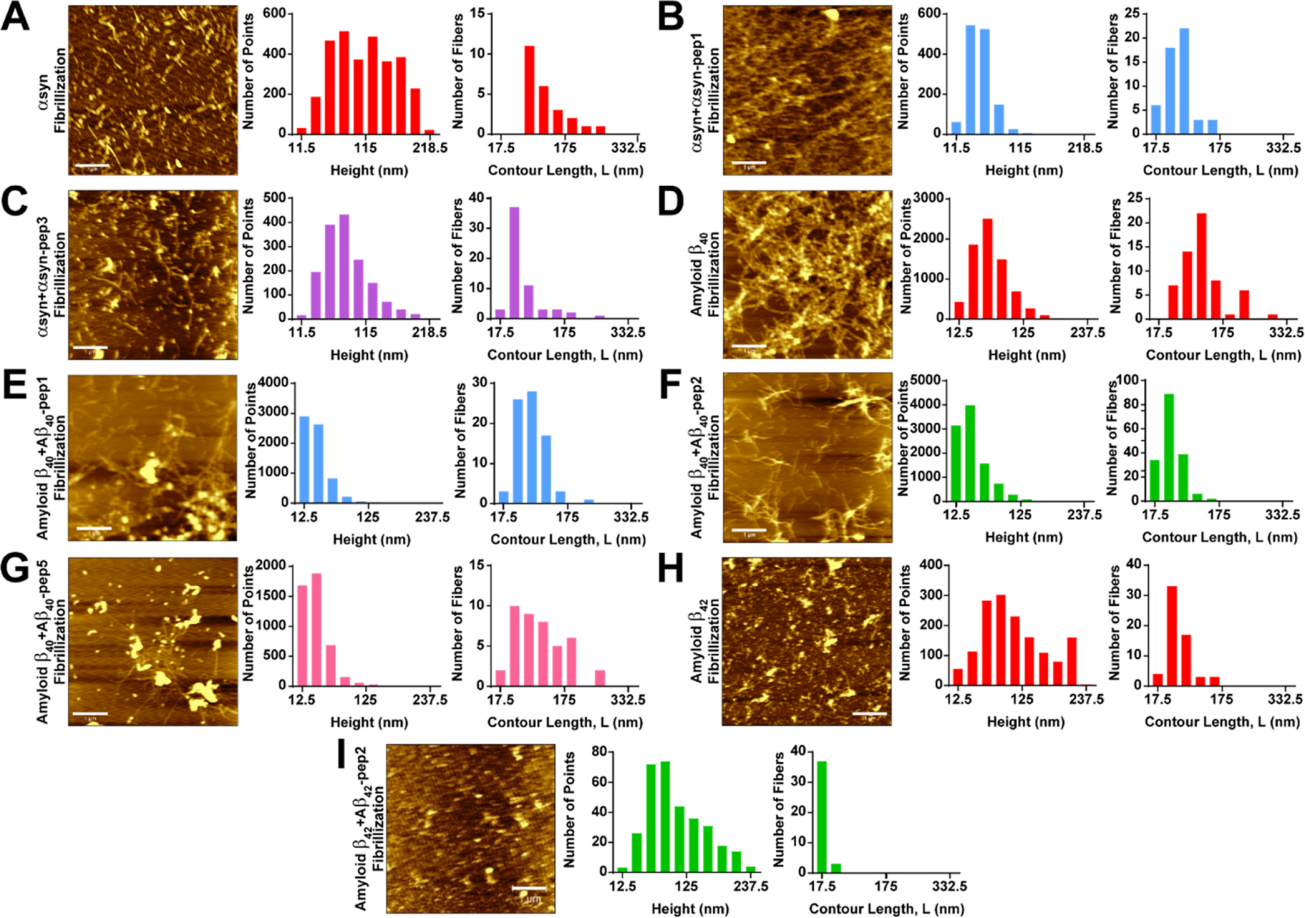
Fibrillization states of monomeric NDPs with and without peptides were analyzed by AFM in tapping mode under dry conditions to detect fibrillization states. For the analysis of the fibrils, FiberApp was used. (A) α-Synuclein fibrillization was achieved without any peptides. The heights and lengths were analyzed from detectable fibrils. (B, C) α-Synuclein with αsyn-pep1 and αsyn-pep3 fibrillization assay was analyzed, and heights and lengths from distinct fibrils were determined individually. (D) Amyloid β_40_ fibrillization product without any peptide addition was analyzed, and the heights and lengths of the detectable fibrils were determined. (E–G) Amyloid β_40_ fibrillization products with Aβ_40_-pep1, Aβ_40_-pep2, and Aβ_40_-pep5 were analyzed, and heights and lengths were determined, respectively. (H) Amyloid β_42_ fibrillization was evaluated. The heights and lengths were determined from distinctively observed spikes. (I) Amyloid β_42_ fibrillization products with Aβ_40_-pep2 were visualized by AFM as seeds whose heights and lengths were determined by FiberApp.

The evaluation of peptide interactions and their effects on the fibrillization of NDPs was carried out using AFM topographical analysis. The analysis involved the evaluation of up to 70 individual fibrils using the FiberApp analysis tool. Each distinct fibril or fibril branch (spikes) was evaluated and compared with itself only because the background signals were high in each data due to high and nonuniform aggregation and fibrillization.^38^ The AFM determined more coherent heights, but the comparison of heights and contour lengths was determined by FiberApp. The results of the analysis are presented in Figures 4 and S6. AFM analyses were conducted by using only NDP fibrils and mixtures of peptides with NDP monomers that were incubated for fibril formation. To evaluate QCM data further, we tried to exhibit the effect of peptides on fibrillization processes by AFM.

The analysis of α-synuclein fibrillization revealed various profiles for both α-synuclein fibrils alone and in mixtures with α-synuclein inhibitory peptides. AFM images of α-synuclein fibrillization showed the presence of individual seeds and non-identical fibril branches of varying sizes (Figure 4A). The seeds and fibril spikes were distinguishable and easily countable. Their intensities were relatively high, indicating large volume sizes. The height and contour length (*L*) of all detectable α-synuclein fibrils were analyzed, and there was significant heterogeneity in both parameters due to the state of fibrillar maturation. However, larger and longer fibrils were considered mature α-synuclein fibrils. Comparing α-synuclein fibril AFM data with α-synuclein + αsyn-pep1 fibrillization, the latter occurred in a crowded mesh-like structure with lower intensities and shorter calculated heights and detectable individual fibril spike lengths (Figure 4B). Despite this, the mesh-like fibril structures made it difficult to detect separate and individual fibrils, indicating αsyn-pep1’s molecular interaction capacity for α-synuclein by increasing fibrillization. Thus, such fibril formation demonstrated that αsyn-pep1 is not a good candidate for aggregation inhibitory molecule by producing intense fibril structures.

Conversely, AFM image data for α-synuclein + αsyn-pep3 fibrillization showed a positive inhibitory effect on fibrillization, with a relatively higher seed amount and fewer fibril branches (Figure 4C). Although the lengths of the fibrils were diverse, the contour lengths were generally short, indicating αsyn-pep3’s inhibitory effect on fibrillization. When compared with αsyn-pep1, αsyn-pep3 appears to be a good candidate as an aggregation inhibitory molecule.

When the fibrillization of α-synuclein with αsyn-pep2, αsyn-pep4, and αsyn-pep5 was analyzed individually, AFM image data showed mesh-like fibrillization with diverse fibril intensities and short detectable individual fibril lengths (Figure S6A–C). These peptides created intense fibril structures, even for fibrils observed in only α-synuclein fibrillization product. Although the aggregation mesh created by these fibrils would be long, these were not distinguishable as single fibrils. Thus, these three peptides were also considered as interacting peptides with positive effects on fibrillization rather than considering as aggregation inhibitory molecules. In addition to AFM analyses, dot-blot assay and end-point Thioflavin-T (ThT) fluorescence measurements were also done to support this evaluation. For dot-blot assay, the samples analyzed with AFM were used. The comparison was done for monomeric unit existence. When monomers were utilized for fibril formation, the dot intensity was expected to be more faint according to control which was only monomeric NDP. As observed in AFM results, α-synuclein + αsyn-pep3 exhibited more intense dot as observed in monomeric α-synuclein (Figure S7A). α-Synuclein + αsyn-pep4 and α-synuclein + αsyn-pep5 exhibited faint dots as closer to α-synuclein fibril and α-synuclein fibril + seed dots. In the case of Tht analysis, α-synuclein + αsyn-pep5 exhibited more fluorescence, which indicates that αsyn-pep5 had a higher impact on fibrillization with higher ThT fluorescence than α-synuclein fibrillization with ThT fluorescence. On the other hand, αsyn-pep3 had the lowest ThT fluorescence than monomeric α-synuclein since it inhibited fibril formation (Figure S8A).

According to AFM analyses, fibrillization of amyloid β_40_ and amyloid β_42_ separately showed different fibrillization profiles. Amyloid β_40_ alone exhibited proper fibrillization profiles with mesh-like fibrils having seeds in the fibril branch centers (Figure 4D). Although detecting individual fibrils was challenging, the selected fibrils were relatively higher in height and length as expected. For the fibrillization of amyloid β_40_ + Aβ_40_-pep1, there were short fibril spikes with diverse-sized amyloid aggregates (Figure 4E). According to the fibril intensities, the aggregates were relatively less intense, and the fibril sizes possessed considerably shorter lengths with less thickness than amyloid β_40_ fibrils. Hence, Aβ_40_-pep1 is a good candidate for blocking fibril formation. The amyloid β_40_ + Aβ_40_-pep2 AFM data also showed decreased fibrillization with shorter and less amount of fibril spikes (Figure 4F). This result demonstrated Aβ_40_-pep2 as a promising blocking aggregation molecule candidate. Also, seed aggregates were observed in fewer amounts over the whole fibrillar structures. Although detectable branches were high in amount, most possessed small contour lengths, and heights were prone to be small. When the fibrillization of amyloid β_40_ + Aβ_40_-pep5 was analyzed, seeds dominated the fibrillization profile which confirmed that Aβ_40_-pep5 could be used as an aggregation inhibitory peptide but it required more analyses for proof of the effect (Figure 4G). Fibril branches were highly separate and individually countable, and long as observed in amyloid β_40_ fibrils, although the amount was low. The contour lengths were diverse, and heights tended to be less in amount. The overall profile of amyloid β_40_ + Aβ_40_-pep5 mix did not fit or show complete similarities with either fibrillization profiles or inhibited fibrillization. The existence of intense and large volumes of seeds and long and separate fibrils exhibited more atypical fibrillization than the others. From amyloid β_40_ + Aβ_40_-pep3 and amyloid β_40_ + Aβ_40_-pep4 fibrillization AFM image data, fibrils obtained in the existence of Aβ_40_-pep3 were less crowded than those with the existence of Aβ_40_-pep4 (Figure S6D,E). However, according to the data, Aβ_40_-pep3 and Aβ_40_-pep4 had less positive effects on the inhibition of aggregation and fibrillization.

Further, dot-blot and ThT fluorescence measurements were performed. According to the dot-blot assay, amyloid β_40_ monomers and fibrils produced intense dots (Figure S7B). Also, except amyloid β_40_ + Aβ_40_-pep1, all amyloid β_40_ and peptide mixtures produced intense dots. The reason might be because of blocking the interaction of the antibody with monomeric units of amyloid β_40_. Besides the dot-blot assay, ThT fluorescence emission results showed that amyloid β_40_ + Aβ_40_-pep5 produced a higher signal than amyloid β_40_ fibril alone and with the peptide mixtures. However, amyloid β_40_ + Aβ_40_-pep1 and amyloid β_40_ + Aβ_40_-pep2 fibril samples produced higher signals than amyloid β_40_ + Aβ_40_-pep3 and amyloid β_40_ + Aβ_40_-pep4 mixtures as well as the monomeric amyloid β_40_. The reason for this might be due to the blockage of cross β structures, where ThT binds, by peptides (Figure S8B).

AFM analysis showed that fibrillization inhibition of amyloid β_42_ had an effect on the growth of peptide fibrils (Figure 4H). In contrast to amyloid β_40_ fibrils, amyloid β_42_ fibrils produced fewer mesh-like fibrillar structures, with the dominant structure being seeds in high amounts. Considerable spikes originating from seeds were also observed, along with individual fibrils, though the lengths of the fibrils were shorter than those observed in cases of amyloid β_40_ and α-synuclein. Nevertheless, fibril growth was initiated and showed immature amyloid fibril structures. The product of amyloid β_42_ + Aβ_42_-pep2 gave closer seed number compared with the amyloid β_42_ fibrillization product. Even though the molecular interaction capacity was high for Aβ_42_-pep2, the inhibition effect of seed or fibril formation was less than expected (Figure 4I). Fibrillization of amyloid β_42_ with Aβ_42_-pep1 resulted in fewer seeds and no fibrillar structure (Figure S6F). Seeds had no branching or spike-like structures, and their sizes were diverse, with higher heights and short aggregate lengths. Similarly, amyloid β_42_ + Aβ_42_-pep3 produced less seed amounts than only amyloid β_42_ fibrillization and amyloid β_42_ + Aβ_42_-pep1 fibrillization product (Figure S6G).

Even though the length of the amyloid β_42_ units had similar values in the product of the amyloid β_42_ + Aβ_42_-pep2 mixture, some of the seed structures were prone to branching. The height distribution was in a wide range for the detectable spike-like structures. To determine fibril formation depending on β-sheet structures, dot-blot assay and ThT fluorescence emissions were measured. Amyloid β_42_ monomers exhibited the most intense dot (Figure S7C). Also, amyloid β_42_ fibrils produced more intense dot than expected. On the other hand, the amyloid β_42_ + Aβ_42_-pep3 mixture resulted in a less intense dot. The differences in the intensity and AFM results might be due to the disruption of antibody interaction with monomeric amyloid β_42_. For ThT measurements, fibrils produced only from amyloid β_42_ emitted more fluorescence than the rest, followed by amyloid β_42_ + Aβ_42_-pep1. Monomers of amyloid β_42_ produced more ThT fluorescence signal than amyloid β_42_ + Aβ_42_-pep3 and amyloid β_42_ + Aβ_42_-pep2, respectively (Figure S8C). The higher signal from amyloid β_42_ + Aβ_42_-pep3 was expected, and it was predicted that amyloid β_42_ + Aβ_42_-pep1 would possess a lower ThT signal relatively than the other amyloid β_42_ + Aβ_42_-pep mixtures.

The reason for the unexpected results can be attributed to several factors. First, Aβ_42_-pep3 may have blocked the β-sheets that ThT interacts with, resulting in lower ThT fluorescence signals. Although fibrils were observed in the amyloid β_42_ + Aβ_42_-pep3 samples, the presence of Aβ_42_-pep3 may have interfered with the ThT detection. Additionally, the QCM results indicated that the molecular interaction capacity of Aβ_42_-pep3 compared to amyloid β_42_ was low, which may have contributed to the reduced ThT signal. Second, Aβ_42_-pep1 may have intrinsic fluorescence, leading to higher fluorescence signals despite the absence of fibrillization observed in AFM analysis. Although the mass accumulation amount of Aβ_42_-pep1 was low according to QCM results, it may still be useful in designing peptide-based drugs or sensor molecules for detection. Finally, in the case of amyloid β_42_ + Aβ_42_-pep2, the ThT signal was the lowest, possibly due to the fact that Aβ_42_-pep2 had a higher interaction capacity compared to amyloid β_42_, as confirmed by the AFM image showing no distinct fibrils.

The biopanning protocol utilized in our study involved the use of yeast cells displaying neurodegenerative proteins as monomers. This approach allowed for the display of NDP monomers on the surface, along with post-translational modifications, and enabled the selection of peptides based on their expression levels, which increased during the process. Following the selection of phage particles from the library, we obtained peptide sequences, from which we selected five peptides for α-synuclein and amyloid β_40_, as well as three peptides for amyloid β_42_. We then analyzed these peptides using QCM and AFM to evaluate their interaction efficiency and their effect on fibrillization, respectively.

The QCM analysis with the same concentration of peptides showed that some of them showed a gradual decreasing pattern for ΔF values with diverse mass accumulations. αsyn-pep1, αsyn-pep3, Aβ_40_-pep1, Aβ_40_-pep2, Aβ_40_-pep5, and Aβ_42_-pep2 showed high and more gradual mass accumulation than the other peptides. Based on these results, fibrillization production was completed for all monomeric units with and without peptides. The fibrillization products showed the amyloid oligomerization and fibrillization states and the effect of peptides on the fibrillization event. αsyn-pep3, Aβ_40_-pep1, Aβ_40_-pep2, Aβ_40_-pep5, Aβ_42_-pep1, and Aβ_42_-pep2 had positive effect on fibrillization which indicates that they cannot be used as aggregation inhibition molecules. However, the dot-blot assay for Aβ_40_-pep1, Aβ_42_-pep2, and Aβ_43_-pep3 as well as ThT results for Aβ_40_-pep1, Aβ_40_-pep2, and Aβ_42_-pep1 conflicted with the fibrillization analysis by AFM. Still, each of the peptides had an effect on fibril formation by promoting or inhibiting the process.

Overall, peptide interaction studies should be evaluated with several approaches for such fibril-forming proteins. The size differences and the interaction capacities between peptides and aggregative proteins should be evaluated in different approaches. In this study, we tried to evaluate the selected peptides with powerful analyses to prove our concept as advantageous and easy to apply. Besides the methodologies used in this study, fibril-forming protein–peptide interaction can also be evaluated in cell culture for further verification. Whether or not, the yeast surface display and phage display library combination with biopanning protocol provides highly interacted peptides for further use. In our case, briefly, αsyn-pep3, Aβ_40_-pep1, Aβ_40_-pep2, and Aβ_42_-pep2 are good candidates for peptide-based drug developments for blocking fibrillization from the early stages of the disease conditions.

Further, αsyn-pep1, αsyn-pep2, αsyn-pep4, αsyn-pep5, Aβ_40_-pep3, Aβ_40_-pep4, Aβ_40_-pep5, Aβ_42_-pep1, and Aβ_42_-pep3 can be engineered and utilized as sensor molecules for detecting the disease conditions. For the detection, the over-expression of amyloid units can interact more with the sensor candidate peptides and give higher intense fibril formation as well as ThT fluorescence signals.

The combination of yeast surface display and phage display library has high potential for selecting peptides for any type of aggregation-prone proteins by expressing as monomeric units. The advantages of this approach are stabilization and expression of nonstable proteins on the yeast surface. Also, the application is relatively easy for such targets rather than immobilizing them onto a solid surface. Still, the combination of yeast surface display and phage display library takes time to prepare the target because of the growth rate of yeast cells. Also, there is a chance to select only interacting peptides without any effect on the inhibition of the problematic processes. Although there is a chance of selecting nonspecific peptides, increasing the number of preselection of phage display library for obtaining more specific library for further use and increasing the number of biopanning cycles can help overcome such disadvantage. In addition, the selected peptides can be computationally analyzed for interaction with the target for obtaining more powerful molecule for the purposes, and they can be used for antibody or nanobody engineering by using the selected peptides as epitope tags.^39^

Hence, the peptides selected and analyzed in our study showed a significant impact on amyloid fibril formation, highlighting the potential of our yeast surface display and phage display library approach in discovering peptide-based therapeutics and molecular diagnostic tools for neurodegenerative diseases. The simple application protocol, high yield of displayed proteins, and short application time of this combination make it a promising approach for developing new drug molecules or detection platforms. Since early diagnosis and halting disease progression are crucial in treating Parkinson’s and Alzheimer’s diseases, the use of surface display platforms to select novel peptide molecules holds great promise in diagnosing and blocking these diseases.

## Methods

### Materials

For surface display, *S. cerevisiae* strain EBY100 (ATCC MYA-4941) (a GAL1-AGA1::URA3 ura3–52 trp1 leu2Δ1 his3Δ200 pep4::HIS2 prb1Δ1.6R can1GAL) was purchased. Ph.D.-12 Phage Display Peptide Library Kit (New England BioLabs Inc., MA) was preferred in order to select aggregation inhibitory peptide selection. For in vitro characterization studies, amyloid β_40_ and amyloid β_42_ were purchased (Genscript, Cat.#RP10004 and #RP10017, respectively). For the interaction studies, QCM-D gold chips were purchased from Biolin Scientific (QSX 301).

### Construction of Plasmids

Yeast surface display constructs were done by using pETcon (−) plasmid, which was a gift from Andrew Scharenberg (Addgene plasmid # 41522; http://n2t.net/addgene:41522; RRID:Addgene_41522). Amyloid β_40_ and amyloid β_42_ genes were extended and amplified by overlap extension PCR, and cloned by the Gibson assembly method in the pETcon (−) plasmid. α-Synuclein gene was synthesized as a fragment gene by Genewiz (Genewiz, NJ) and cloned in pETcon (−) plasmid by the Gibson assembly method. For protein expression and purification in *E. coli*, α-synuclein was amplified by PCR. As the backbone plasmid, pET22b-6h-GST-TEVp was digested with *Bam*HI and *Xho*I restriction enzymes in order to remove the TEVp gene (Addgene plasmid #172887). α-Synuclein was cloned into the pET22b-6h-GST backbone by Gibson assembly method. All cloned constructs were chemically transformed into *E. coli* DH5α PRO strain. All constructs were verified by Sanger sequencing (Genewiz, NJ).

### Transformation of EBY100 Cells

*S. cerevisiae* strain EBY100 (ATCC MYA-4941) (a GAL1-AGA1::URA3 ura3–52 trp1 leu2Δ1 his3Δ200 pep4::HIS2 prb1Δ1.6R can1GAL), Trp-Leu- was utilized for the surface expression of α-synuclein, amyloid β_40_, and amyloid β_42_. pETcon constructs were transformed into the EBY100 cells via electroporation. Electrocompetent EBY100 cells were prepared following the protocol developed by Suga and Hatakeyama.^40^ Briefly, EBY100 cells were grown overnight to reach a cell density value of around 1 × 10^7^ cells/mL. Cell culture was placed on ice for 15 min and centrifuged at 4000*g* for 5 min. Pellet was washed three times in cold sterile double-distilled water. After washing steps, cells were resuspended in ice-cold freezing buffer (2 M sorbitol, 10 mM CaCl_2_ and 10 mM HEPES (pH 7.5)) as the cell concentration became 5 × 10^8^ cells/mL. 100 μL aliquots of cell suspension were transferred into the 1.5 mL sterile cryotubes. After thawing electrocompetent cells in a 30 °C water bath, 1 mL of 1 M sorbitol was added. Cells were centrifuged and the pellet was dissolved in 1 M sorbitol to adjust the cell density to 1–2 × 10^8^ cells/mL. After the addition of purified plasmid, cell suspension was transferred into a cuvette with a 2 mm gap size. Following the electrical pulse (voltage: 2 kV, resistance: 200 Ω, capacitance: 25 μF), 1 mL of ice-cold sterile 1 M sorbitol was immediately added onto the cells. Next, 100–200 μL of the cell mixture was spread onto the synthetic drop-out (SD)-agar plates to be incubated at 30 °C for 2–3 days.

### Induction of Yeast Cells for Surface Display

In order to induce surface display of the neurodegenerative disease-related proteins, EBY100 cells were first grown in 3–5 mL of synthetic drop-out media without tryptophan including 2% dextrose (SD-CAA) with constant shaking at 260 rpm at 30 °C. The OD_660_ value was measured and cells were transferred into fresh SD-CAA and the cell density was arranged to be approximately 4.6 × 10^6^ cells/mL. The concentration was sufficient to reach an OD_660_ value of 1 in 4-h incubation at 260 rpm at 30 °C. After incubation, the cell suspension was centrifuged at 4000*g* for 5 min and the cell pellet was dissolved in selective induction medium (1.92 g/L synthetic drop-out medium without tryptophan, 6.7 g/L yeast nitrogen base without amino acids, 2% galactose, 0.2% glucose, 1× phosphate buffer, and 100 μg/mL ampicillin). Cells within the synthetic drop-out induction medium with galactose (SD-GAA) were incubated at 260 rpm at 20 °C for 16–20 h.

### Immunostaining of Yeast Cells

1 × 10^6^ of induced yeast cells were centrifuged and induction medium was discarded. After washing the cell pellet with 1× PBS, cells were resuspended in 1 mL of blocking solution (1% BSA in 1× PBS [w/v]). For blocking, the cells were incubated at room temperature for 2 h. At the end of the blocking incubation, cells were centrifuged and blocking solution was discarded. Then, cells were washed three times with 1× PBS. After washing, cells were resuspended in 250 μL 1% BSA in 1× PBS containing Myc-tag Mouse monoclonal antibody (Cell Signaling, 9B11, Cat.#2276) with a ratio of 1:500. Primary antibody incubation was done at room temperature for 3 h. Following the primary antibody incubation, cells were washed with 1× PBS three times. Then, cells were resuspended with 250 μL 1% BSA in 1× PBS containing 1:500 Goat Anti-Ms IgG (H + L) Cross-Adsorbed Secondary Antibody, DyLight550 conjugate (Thermo Scientific, SA5-10173). The secondary antibody incubation was done at room temperature for 3 h. At the end of the incubation, cells were washed with 1× PBS three times and resuspended in 250 μL of 1× PBS. The labeled cells were placed on positively charged slides and examined using a Zeiss Axio Scope.A1 microscope.

### Biopanning against Yeast Expressing Neurodegenerative Proteins on the Surface

To select aggregation inhibitor peptides, yeast surfaces displaying NDPs were produced as explained in the section on EBY100 induction. Ph.D.-12 Phage Display Peptide Library Kit (New England BioLabs Inc., MA) was amplified following the recommended protocols by the manufacturer. Before selecting ligand peptides, the negative selection was done by using Aga2p displaying EBY100 cells. Unbound phages were eluted and amplified as specified in the manual of the library. The unbound phage library was used to select peptides against EBY100 cells displaying NDPs. For all biopanning methods (for both negative selection and the selection), 10^11^ yeast cells were used at the beginning of each biopanning cycle. All centrifugation steps for yeast cells were done at 3000*g* for 3 min. The number of phage particles used in each cycle of biopanning was 10^9^. Except for the negative selection, bound phages were eluted and amplified for further biopanning cycles. Each biopanning cycle was done by using Protein LoBind Tubes (Eppendorf) in order to avoid nonspecific interactions of proteins with the tubes during biopanning. At the beginning of each cycle, 10^11^ yeast cells were centrifuged down and the pellet was washed with 1 mL of 0.1 M NaHCO_3_, pH 8.6 coating buffer. After centrifugation, the pellet was again resuspended with 1.5 mL of the same buffer and incubated at +4 °C overnight onto the rotator. Next day, cells were centrifuged and the coating buffer was removed. The cell pellet was washed with 1.5 mL of 1× TBS-T and centrifuged. Then, the pellet was resuspended with 1.5 mL of 0.1 M NaHCO_3_, pH 8.6 containing 5 mg/mL BSA, and incubated for 2–4 h at 4 °C on the rotator. At the end of the blocking, cells were centrifuged and washed 6 times with the 1× TBS-T buffer. When wash steps were finished, 10^9^ phage particle was added onto the cell pellet and the volume was completed to 1 mL with 1× TBS. For achieving interaction, cells and phages were incubated at room temperature for 1 h on the rotator for mixing them thoroughly. At the end of phage-binding incubation, cells were centrifuged and washed 10 times with 1.5 mL of 1× TBS-T to get rid of the unbound phages. For elution of the bound phages, 1 mL of 0.2 M glycine-HCl, pH 2.2 elution buffer, was added to cells and incubated at room temperature for 7 min with gentle shaking. To get the phage elution, the supernatant was transferred to a new tube and 150 μL of neutralization buffer (1 M Tris-HCl, pH 9.1) was added.

### Phage Titering

Overnight culture of *E. coli* ER2738 cells was diluted with a ratio of 1:100 into LB containing tetracycline antibiotic. When the OD_600_ value reached 0.5, 200 μL of cells were transferred into sterile tubes. Meanwhile, amplified phages and eluted phages were diluted with a dilution factor of 10^–8^–10^–12^ and 10^–2^–10^–4^, respectively. For each dilution, 10 μL of samples were taken and added to 200 μL of cells. Phages were incubated with cells for 1–5 min at room temperature in order to infection occurred. At the end of incubation, cell-phage samples were mixed with 3 mL of top agar at 45 °C. Top agar containing phage–cell mixture, then, was spread onto LB agar plates containing X-Gal, IPTG, and tetracycline. The plates were incubated at 37 °C overnight to complete blue/white screening. Next day, the number of plaques was counted to calculate the enrichment ratio.

### Eluted-Phage and Single-Phage Plaque Amplification

To use eluted phages for the next round of biopanning, overnight culture of *E. coli* ER2738 was diluted with a ratio of 1:100 into 20 mL of LB containing tetracycline. The diluted culture was incubated until the OD_600_ reached 0.01–0.05. After getting the appropriate OD_600_ value, eluted phages were added onto the culture, and incubation was done at 37 °C, 200 rpm for 4.5 h to propagate phage elution. Then, the culture was centrifuged at 12 000 rpm, 4 °C for 10 min. The supernatant having amplified phages was transferred into a new tube; 5 mL of 20% PEG-8000/2.5 M NaCl solution was added to phage precipitation after overnight incubation at 4 °C. Next, phages were centrifuged at 12 000 rpm, 4 °C for 15 min. The supernatant was discarded and the finger-like pellet was resuspended with 1 mL of 1× TBS. Additional phage precipitation was applied by the addition of 200 μL of 20% PEG-8000/2.5 M NaCl solution. The phage suspension was incubated on ice for 1 h. At the end of the incubation, centrifugation was done at 14 000 rpm, 4 °C for 5 min. The phage titering was done for this amplified phage solution. For using a single-phage clone for further experiments, the single-phage plaque was selected with a tip and inoculated to 1 mL of *E. coli* ER2738 culture at OD_600_ value between 0.01 and 0.05, which was prepared with overnight culture as diluted with a ratio of 1:100 into 20 mL of LB containing tetracycline. The amplification in the 1 mL culture was carried out at 37 °C for 4.5 h at 200 rpm. Then, the cell culture was centrifuged at 14 000 rpm for 30 s. The upper 800 μL of supernatant was taken gently without disrupting the cell pellet. Then, 20 μL from the 800 μL single-phage suspension was further amplified by following the same protocol for eluted-phage amplification. All phage suspensions were stored at 20 °C with 50% glycerol (1:1 v/v) for long term.

### Expression and Purification of α-Synuclein

For protein purification, pET22b-α-synuclein-TEV-GST-6H plasmid was transformed chemically into the *E. coli* BL21 (DE3) strain. Single colony for α-synuclein expression was picked and inoculated into 1 L of autoinduction media.^41^ Overnight grown and induced cells having pET22b-α-synuclein-TEV-GST-6H plasmid were harvested for purification steps. Cells were centrifuged at 8000*g* for 5 min. Next, the pellet was resuspended in 100 mL of lysis buffer (20 mM imidazole, 500 mM NaCl, 20 mM sodium phosphate, pH 7.4). A 1 mL of 100 μL PMSF was added to the cell suspension. To lyse the cells, first, liquid nitrogen freezing and thawing were applied five times. Then, sonication was done by applying 800–900 kJ energy with 30% power for about 2 min of 10 s on/10 s off. After lysis of the cells, centrifugation was done at 15 000*g* for 30 min. The lysate was transferred to a fresh tube after being filtered with 0.45 μm syringe cellulose filters. The protein purification was achieved by using the HisTrap nickel column (GE Life Sciences 17524701) for FPLC (ÄKTA start protein purification system) as per the manufacturers’ specified protocol. At the end of the protocol, the elutions having α syn-GST protein were collected (Figure S2).

### Producing Monomers of α-Synuclein

To get the monomeric version of α-synuclein, purified α-syn-TEV Recognition Site-GST-6His protein was transferred to the TEV reaction buffer (50 mM Tris-HCl [pH 8.0], 0.5 mM EDTA, and 1 mM DTT). TEV protease reaction was done by using 500 μg/mL α syn, 100 μg/mL homemade TEV protease-GST-6His, 0.02% sodium azide, and 0.1 mM DTT at room temperature overnight. The monomeric proteins were then purified by using the HisTrap nickel column (GE Life Sciences 17524701) for FPLC (ÄKTA start protein purification system) as per the manufacturers’ specified protocol. The unbound proteins were collected since α-synuclein had no tag to be captured by the HisTag column except TEV Protease-GST-6His and GST-6His from α-syn-TEV Recognition Site-GST-6His. The monomeric α-synuclein was transferred to 1× PBS and phosphate buffer for QCM-D analysis and fibrillization assay, respectively.

### Fibrillization Assay for α-Synuclein, Amyloid β_40_, and Amyloid β_42_

To produce seeds and check the peptide–protein interaction, 100 μM monomeric α-synuclein in phosphate buffer, pH 6, was incubated with and without 10 μM peptides separately in a Grant PCMT Microtube/microplate Thermo-Shaker at 40 °C with 850 rpm for 72 h. For further producing fibrils of α-synuclein, 300 μM monomers in phosphate buffer, pH 6, were incubated with 100 μM seeds and the mix was incubated at 40 °C with 850 rpm for 72 h.

For the fibrillization of amyloid β_40_ in 0.1 M sodium bicarbonate buffer, pH 9, and amyloid β_42_ in PBS, pH 7.4, 100 μM proteins were added into PBS containing 2% DMSO. To increase the fibrillization, HCl was added to attain the 20 mM final concentration of HCL. The fibrillization was achieved after 24 h incubation at 37 °C. Monomers were also incubated with 250 μM peptides separately.

### SDS-PAGE and Western Blotting

For SDS-PAGE and western blot analysis, protein samples were heated at 95 °C with a 1× SDS loading dye. The gel electrophoresis was done with 15% SDS-polyacrylamide gel. After the run was completed, the gel was stained with Coomassie blue staining solution by heating in a microwave oven for 30 s and then being shaken for 5 min at room temperature. The stained gel was then placed in the destaining solution (60% ddH_2_O, 30% methanol, and 10% acetic acid) until the excessive dye was removed and bands were visible. For western blot analysis, the samples in the gel were transferred to the PDVF membrane by the Trans-Blot Turbo (Bio-Rad) system. The membrane was blocked with 5% milk in TBS-T for 1 h at room temperature. After blocking, the membrane was incubated in 5% milk in TBS-T containing 1:5000 primary antibodies for 1 h at room temperature. The membrane was washed with 1× TBS-T and incubated with horseradish peroxidase (HRP)-conjugated secondary antibody for 1 h at room temperature. At the end of incubation, the membrane was washed with 1× TBS-T. Incubation with ECL substrates (Bio-Rad 170-5060) was done to visualize the membrane. The images were taken by Vilber Fusion Solo S. For α-synuclein oligomers, anti-α-synuclein antibody, oligomer-specific Syn33 (Cat.# ABN2265, Millipore), and Goat Anti-Mouse IgG H&L (HRP) (ab6789, Abcam) were used as primary and secondary antibodies, respectively. For HisTag recognition, 6×-His Tag monoclonal antibody (HIS.H8) (MA1-21315, Thermo Scientific) and HRP-conjugated goat anti-mouse secondary antibody (Abcam ab6789-1 MG) were used as primary and secondary antibodies, respectively.

### QCM Analysis

The QCM gold sensor (Biolin Scientific QSense QSX 301 Gold) surface was cleaned, immersed in 20 mM 11-mercaptoundecanoic acid (11-MUA), and activated by EDC/NHS coupling reaction followed by protein immobilization, as previously described.^42^ For each QCM analysis of amyloid β proteins and α-synuclein, 50 and 500 μg of proteins were introduced to the chamber, respectively. After the deactivation of the surface was completed, 1000 μM peptide solution was delivered to the chamber for about 7 min which was followed by 10 min-long washing with 1× PBS. Peptide addition and washing steps were repeated several times in order to analyze the frequency change better. First, third, fifth, seventh, and ninth overtones for frequency values were recorded during the run. Each plot was obtained by depending on the average of Δ*F* values from third, fifth, seventh, and ninth overtones. The mass accumulation was calculated by the formula of Δ*m* = −*C*(Δ*F*/*n*), where Δ*m* is the change in the mass (ng·cm^–2^), *C* is the mass sensitivity constant that depends on the chip specification (17.7 ng·cm^–2^ for QSX 301 gold QCM chip), Δ*F* is the change in the resonance frequency (Hz), and *n* is the number of harmonics. Mass accumulation values were obtained according to the data from the fifth overtone.

### AFM Analysis

Five microliters of fibrils, fibrils with peptides, and monomers were added to the silica wafer in a 96-well plate, and 200 μL of 2.5% glutaraldehyde was added to each wafer. After overnight incubation of wafers at +4 °C, wafers were washed with 1× PBS and then ddH_2_O for 5 min in a shaker at room temperature for three times. Then, the wafers were washed sequentially with 25, 50, and 75% ethanol (EtOH) for 5 min in a shaker at room temperature. Finally, 100% EtOH washing was done for 5 min in a shaker at room temperature three times. After washing, the wafers were first air-dried and argon-gas dried before measuring. AFM images were obtained using an MFP-3D Origin AFM (Oxford Instruments). Diamond-like carbon (DLC)-coated AFM cantilever tip having a spring constant of 40 N/m with a resonance frequency of 300 kHz was preferred. Surface topography of the samples was determined by tapping mode. The sample scanning rate was between 1.2 and 2.2 Hz to minimize the imaging artifacts and the tip–sample interaction.

### Dot-Blot Assay

Five microliters of 10 μM α-synuclein samples and amyloid β samples were dropped onto PDVF membranes. Samples were air-dried. Membranes were placed in a 10 cm petri dish and blocked by 5% BSA in TBS-T for 30 min at room temperature. Next, the membranes were incubated in 5% BSA in TBS-T containing 1:10 000 primary antibodies for 30 min at room temperature. The membranes were washed with 1× TBS-T three times and incubated with horseradish peroxidase (HRP)-conjugated secondary antibody for 30 min at room temperature. At the end of incubation, the membranes were washed with 1× TBS-T. Incubation with ECL substrates (Bio-Rad 170-5060) was done to visualize the membranes. The images were taken by Vilber Fusion Solo S. For α-synuclein monomers, recombinant anti-α-synuclein antibody [ERP20535] (ab212184, Abcam) was used. For amyloid β monomers, β-Amyloid (D54D2) XP Rabbit mAb was used. As secondary antibody, Goat anti-Rabbit IgG (H + L) secondary antibody, HRP (Cat.#31460, Invitrogen), was preferred.

## Supporting Information Available

Figure S1. Yeast surface display expression design and plasmid maps. Figure S2. SDS-PAGE and western blotting results of monomeric α-synuclein purification and fibrillization products. Figure S3. QCM immobilization data of α-synuclein, amyloid β_40_, and amyloid β_42_ for αsyn-pep1, αsyn-pep3, Aβ_40_-pep1, Aβ_40_-pep2, Aβ_40_-pep5, and Aβ_42_-pep2. Figure S4. QCM immobilization and peptide interaction data for α-synuclein + αsyn-pep2, α -synuclein + αsyn-pep4, α -synuclein + αsyn-pep5, amyloid β_40_ + Aβ_40_-pep3, amyloid β_40_ + Aβ_40_-pep4, amyloid β_42_ + Aβ_42_-pep1, amyloid β_42_ + Aβ_42_-pep3, amyloid β_42_ + Aβ_42_-pep4, and amyloid β_42_ + Aβ_42_-pep5. Figure S5. QCM immobilization and negative control peptide interaction with α-synuclein and amyloid β_40_. Figure S6. AFM analysis for fibrillization of α-synuclein + αsyn-pep2, α -synuclein + αsyn-pep4, α -synuclein + αsyn-pep5, amyloid β_40_ + Aβ_40_-pep3, amyloid β_40_ + Aβ_40_-pep4, amyloid β_42_ + Aβ_42_-pep1, amyloid β_42_ + Aβ_42_-pep3, amyloid β_42_ + Aβ_42_-pep4, and amyloid β_42_ + Aβ_42_-pep5. Figure S7. Dot-blot assay for all fibrillization products and monomeric units of α-synuclein, amyloid β_40_, and amyloid β_42_. Figure S8. ThT fluorescence measurements for all fibrillization products and monomeric units of α-synuclein, amyloid β_40_, and amyloid β_42_. Supplementary method for deprotection of resin and coupling with an Fmoc protected amino acid and cleavage reaction during solid-phase peptide synthesis. Supplementary method for ThT assay. Supplementary references (PDF)

## Notes

U.Ö.Ş.Ş., C.E.Ö., and Ö.B. filed and submitted a patent application.
