# Supplementary material for: Synergistic Screening of Peptide-Based Biotechnological Drug Candidates for Neurodegenerative Diseases Using Yeast Display and Phage Display
Source: ACS Chem Neurosci. 2023 Aug 28;14(19):3609–21. doi: 10.1021/acschemneuro.3c00248 (PMC10557061; doi:10.1021/acschemneuro.3c00248)
Supplement: Supplementary file 1 — cn3c00248_si_002.pdf [file cn3c00248_si_002.pdf]

Synergistic Screening of Peptide-Based Biotechnological Drug Candidates for Neurodegenerative Diseases using Yeast Display and Phage Display

Supplementary Materials

Cemile Elif Özçelik[1], Özge Beğli[1], Ahmet Hınçer[1], Mehmet Seçkin Keskin[2], Oğuzhan Oğuz[1], Recep Erdem Ahan[1], Talip Serkan Kasırga[1], Salih Özçubukçu[2], Urartu Özgür Şafak Şeker [1,3]

[1] UNAM- Institute of Materials Science and Nanotechnology, Bilkent University, Ankara, 06800, Turkey

[2] Department of Chemistry, Faculty of Science, Middle East Technical University, Ankara 06800, Turkey

[3] Neuroscience Graduate Program, Bilkent University, Ankara, 06800, Turkey

Contents

Supplementary Figure S1
Supplementary Figure S2
Supplementary Figure S3
Supplementary Figure S4
Supplementary Figure S5
Supplementary Figure S6
Supplementary Figure S7
Supplementary Figure S8
Supplementary Methods
Supplementary References

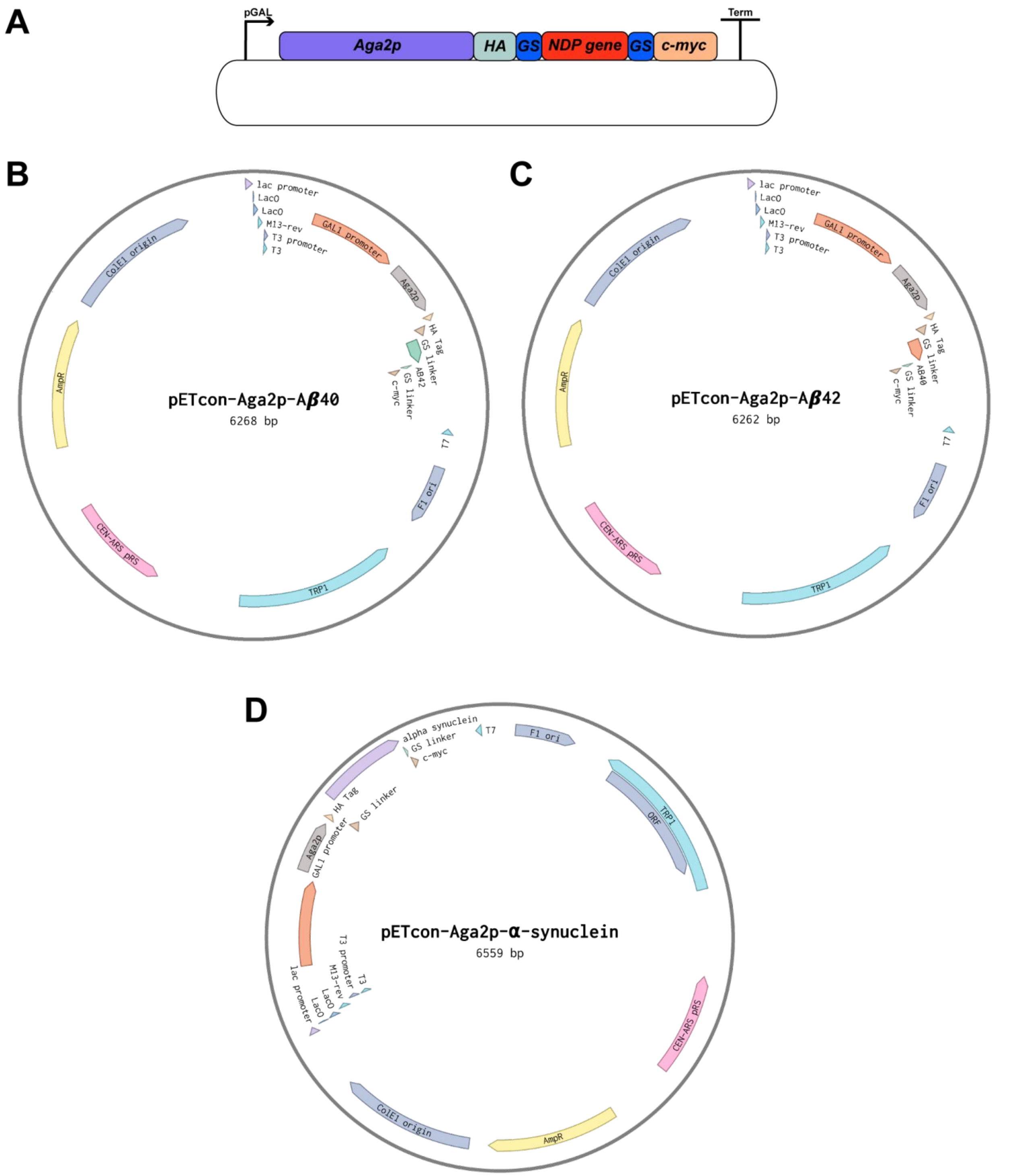

Supplementary Figure S1. The representation of yeast surface display expression cassette and plasmid maps. (A) All NDPs were expressed as a fusion of *Aga2p* gene under galactose-inducible promoter, pGAL. Aga2p and NDP gene were separated by GS linker. HA and c-Myc epitope tags were existed at 3' and 5' of NDP gene, respectively. c-myc tag were used for ICC experiments (B-D) The plasmid maps for pETcon-Aga2p-Amyloid β_{40}, pETcon-Aga2p-Amyloid β_{42}, and pETcon-Aga2p-α-synuclein used in this study were obtained from Benchling[1].

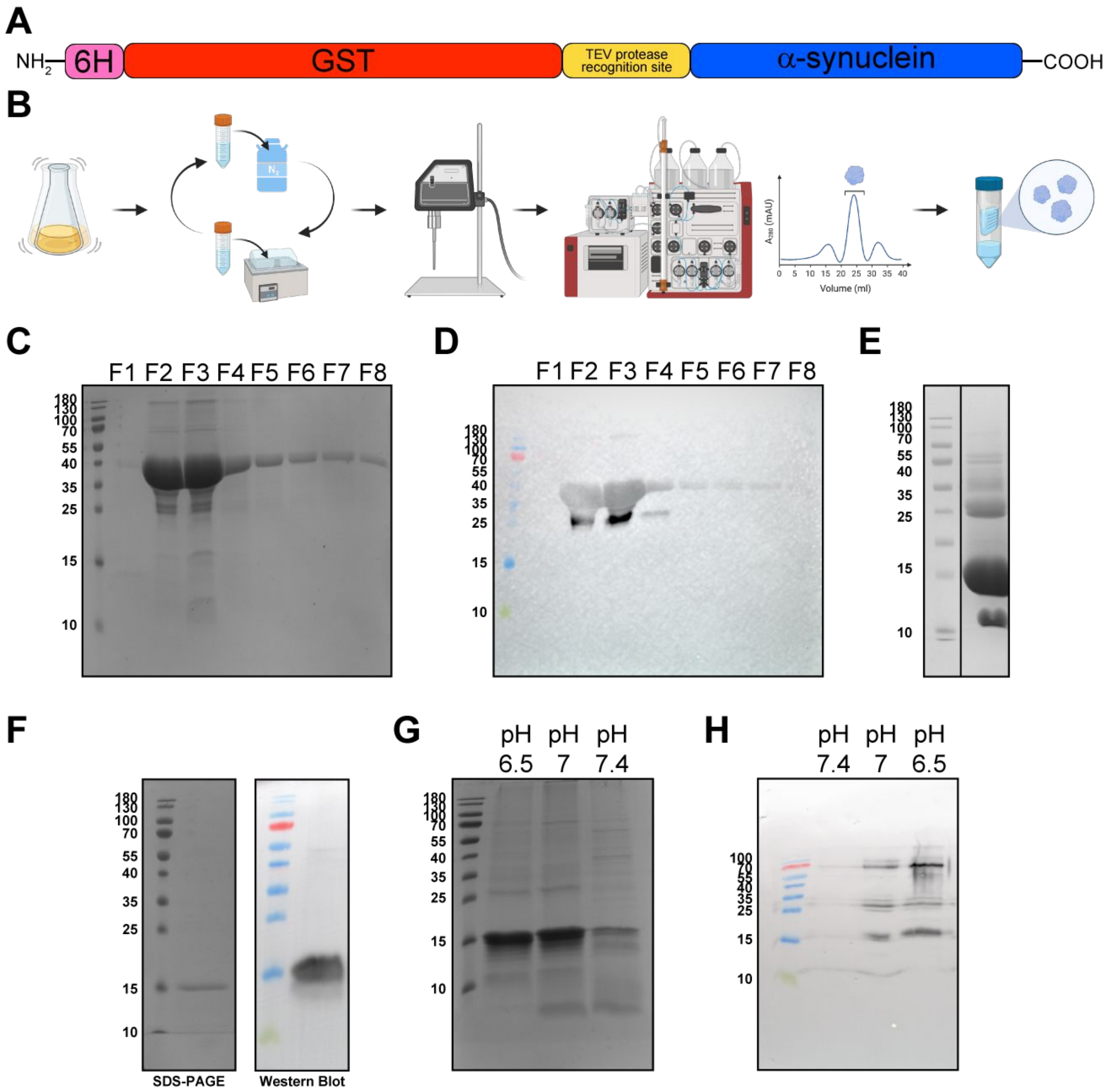

Supplementary Figure S2. α-synuclein monomers and fibrils were prepared by following several steps and purified by FPLC method. First, α-synuclein-GST-6His was expressed *E.coli* BL21 strain by IPTG induction. Cells were lysed by freeze-thaw and sonication. Then HisTag purification was achieved by FPLC. Purified samples were used in TEV protease reaction in order to remove GST-6His region from monomeric α-synuclein. Then, monomeric α-synuclein was purified by FPLC as collecting unbound samples. Fibrillization was done by using PB, pH 6.5 at Thermo-Shaker at 40°C with 850 rpm for 72 hours. All protein samples were analyzed with SDS-PAGE and western blotting. (A) The design of α-synuclein for protein purification. 6H was used for FPLC method, and GST tag was used for solubilization during the expression and purification steps. (B) A simplified workflow for protein purification was represented schematically. The

overnight-induced cells were lysed chemically, which was followed by mechanical lysing with freeze-thaw cycles and sonication. After lysis, FPLC was used for protein purification. Purified proteins were concentrated for TEV protease reaction. For monomeric purification, FPLC step and protein concentration steps were repeated. The figure was created with BioRender.com (C) The fractions obtained after 6H-GST-α-synuclein protein purifications were analyzed by SDS-PAGE. 40 kDa 6H-GST-α-synuclein were obtained in high concentrations in F2 and F3 fractions. (D) Western blotting was done with the same fractions obtained after 6H-GST-α-synuclein purification. (E) Western blotting was done after TEV reaction for cleave 14 kDa monomeric α-synuclein from 6H-GST-α-synuclein. Removed GST and TEVp were observed around 26 kDa and 55kDa, respectively. (F) SDS-PAGE and western blotting were done for the detection of monomeric α-synuclein. (G-H) For the optimization of fibrillization pH, fibrillization assay samples with different pH values were analyzed SDS-PAGE. The monomeric α-synuclein band intensities for samples from PB, pH 6.5, and PB, pH 7, were high. Still, there were low-intensity bands in the upper parts of the gel. (H) Fibrillization assay samples with different pH values were also analyzed by western blotting. Small-sized seeds were detected well in the samples obtained with PB, 6.5 pH.

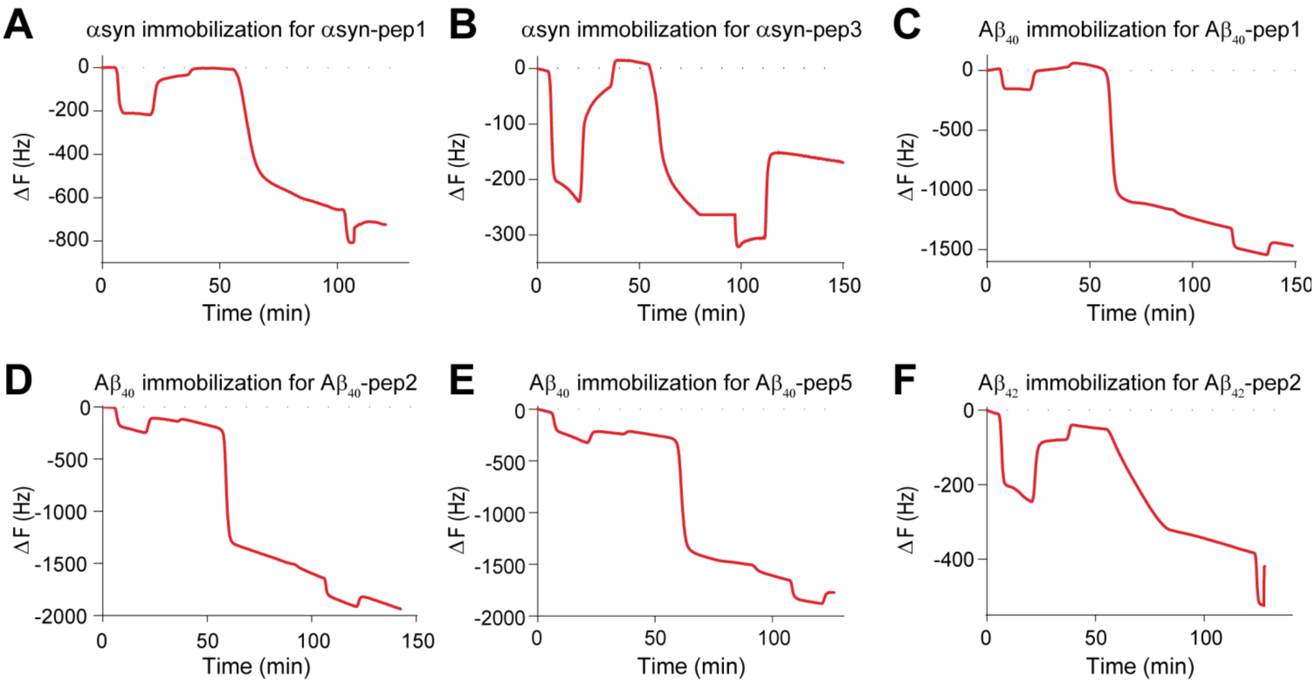

Supplementary Figure S3. The first step of the QCM analysis for NDP-peptide interaction was the immobilization of NDP onto gold chip. The immobilization was achieved by EDC/NHS coupling reaction. Deactivation of the chips were achieved by 1M Ethanolamine HCl. (A-B) Immobilization of α-synuclein for αsyn -pep1, and αsyn-pep3 interactions were achieved with the mass accumulations of 1778 ng.cm^{-2}, and 536 ng.cm^{-2} on the chips, respectively. (C-E) Immobilization of amyloid $β_{40}$ for A$β_{40}$-pep1, A$β_{40}$-pep2, and A$β_{40}$-pep5 interactions were achieved with the mass accumulations of 4536 ng.cm^{-2}, 5090 ng.cm^{-2}, 4648 ng.cm^{-2} on the chips, respectively. (F) Immobilization of amyloid $β_{42}$ for A$β_{42}$-pep2 interaction was achieved with the mass accumulation of1106 ng.cm^{-2}.

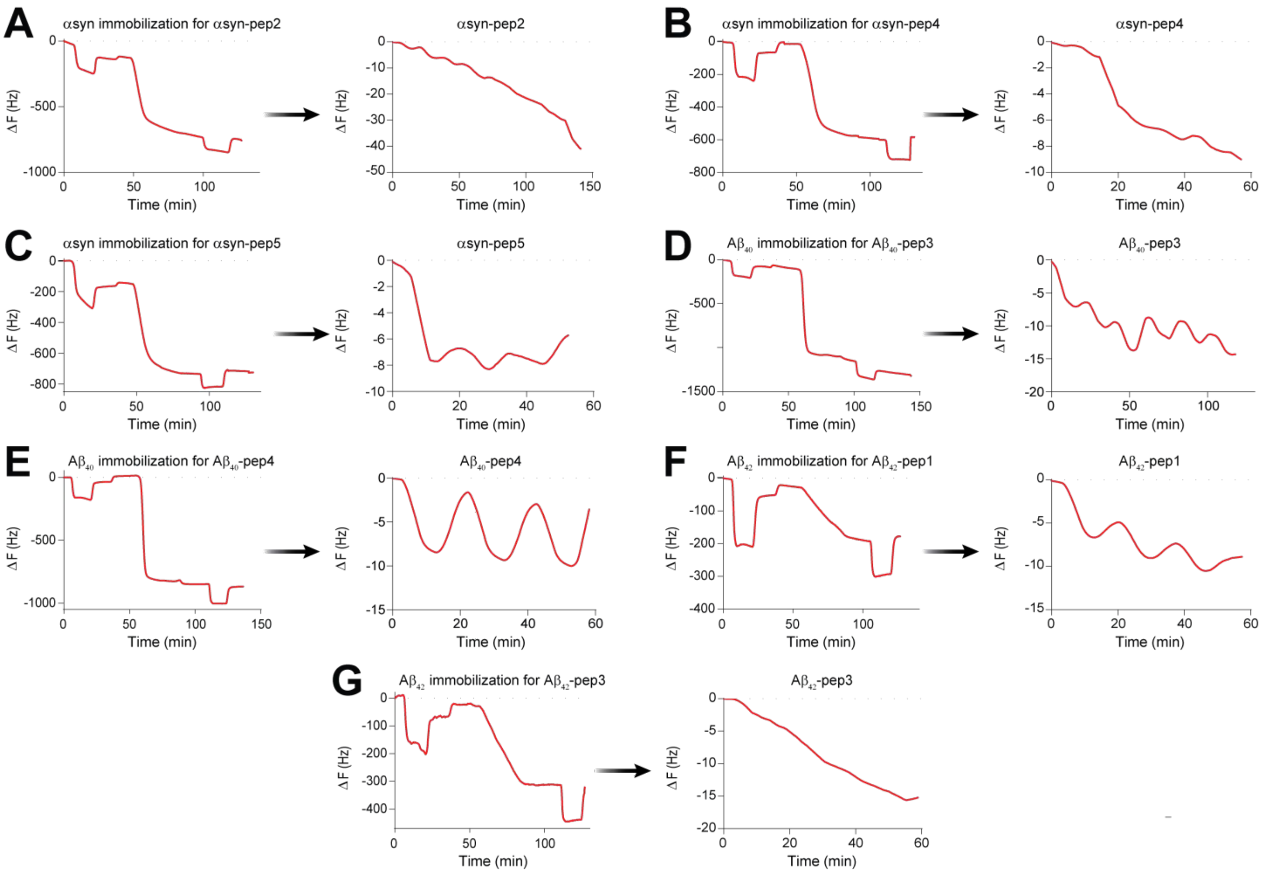

Supplementary Figure S4. For all different peptides for NDPs were analyzed by QCM to determine protein-peptide interaction. First, gold chips were coated with the target NDP monomers by EDC/NHS coupling reaction. This gave the first frequency change graph. Then, peptides were added to QCM chamber flow system to detect whether interaction was occurred or not. (A) Immobilization of α-synuclein for αsyn -pep2 was achieved with the mass accumulations of 1952 $ng.cm^{-2}$ on the chips. The mass accumulation of αsyn -pep2 onto α-synuclein coated gold chip was 427 $ng.cm^{-2}$ (B) Immobilization of α-synuclein for αsyn -pep4 was achieved with the mass accumulations of 1742 $ng.cm^{-2}$ on the chips. The mass accumulation of αsyn -pep4 onto α-synuclein coated gold chip was 27.2 $ng.cm^{-2}$ (C) Immobilization of α-synuclein for αsyn -pep5 was achieved with the mass accumulations of 1776 $ng.cm^{-2}$ on the chips. The mass accumulation of αsyn -pep5 onto α-synuclein coated gold chip was 3.8 $ng.cm^{-2}$. (D) Immobilization of amyloid β_{40} for $A\beta_{40}$-pep3 was achieved with the mass accumulations of 3661 $ng.cm^{-2}$ on the chips. The mass accumulation of $A\beta_{40}$-pep3 onto amyloid β_{40} coated gold chip was 405 $ng.cm^{-2}$ (E) Immobilization of amyloid β_{40} for $A\beta_{40}$-pep4 was achieved with the mass accumulations of 2711 $ng.cm^{-2}$ on the chips. The mass accumulation of $A\beta_{40}$-pep4 onto amyloid β_{40} coated gold chip was 9.5 $ng.cm^{-2}$ (F) Immobilization of amyloid β_{42} for $A\beta_{42}$-pep1 was achieved with the mass accumulations of 2723 $ng.cm^{-2}$ on the chips. The mass accumulation of $A\beta_{42}$-pep1 onto amyloid β_{42} coated gold chip was 134 $ng.cm^{-2}$.(G) Immobilization of amyloid β_{42} for $A\beta_{42}$-pep3 was achieved with the mass accumulations of 856 $ng.cm^{-2}$ on the chips. The mass accumulation of $A\beta_{42}$-pep3 onto amyloid β_{42} coated gold chip was 100 $ng.cm^{-2}$.

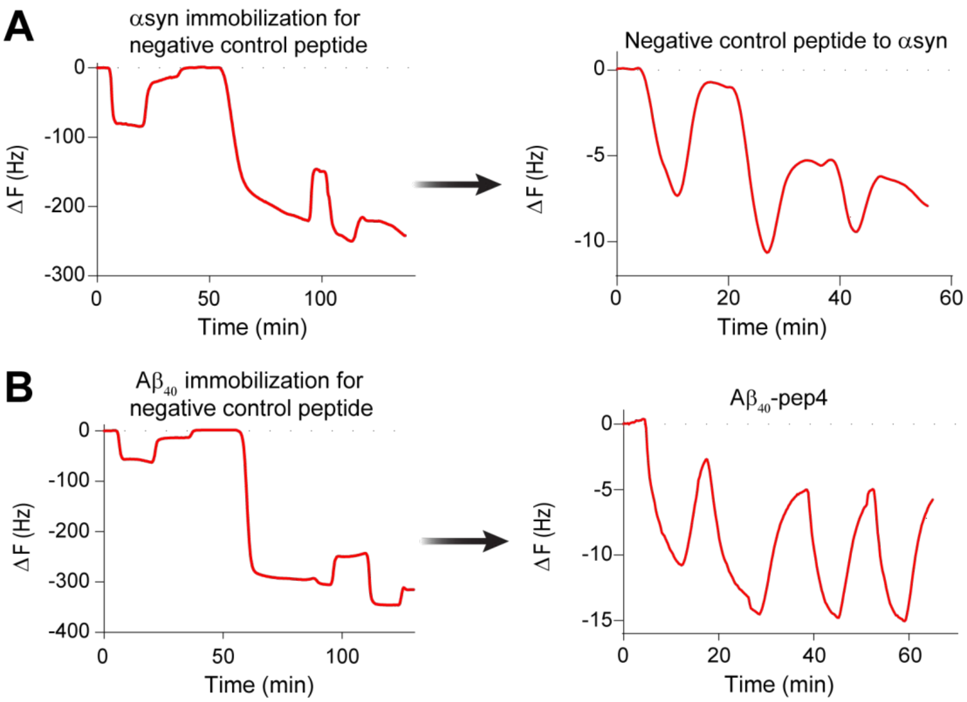

Supplementary Figure S5. Randomly synthesized peptide, KTWMDGFFSYGT, was used as negative control for QCM analysis. The first measurements were for immobilization of 500 µg α-synuclein and 50 µg amyloid β_{42}. The second measurements were for 1000 µM peptide addition to the QCM chamber. (A) Immobilization of α-synuclein for negative control peptide was achieved with the mass accumulations of 655 $ng.cm^{-2}$ on the chips. The mass accumulation of negative control peptide onto α-synuclein coated gold chip was 12.4 $ng.cm^{-2}$ (B) Immobilization of amyloid β_{40} for negative control peptide was achieved with the mass accumulations of 955.8 $ng.cm^{-2}$ on the chips. The mass accumulation of negative control peptide onto amyloid β_{40} coated gold chip was 7.05 $ng.cm^{-2}$.

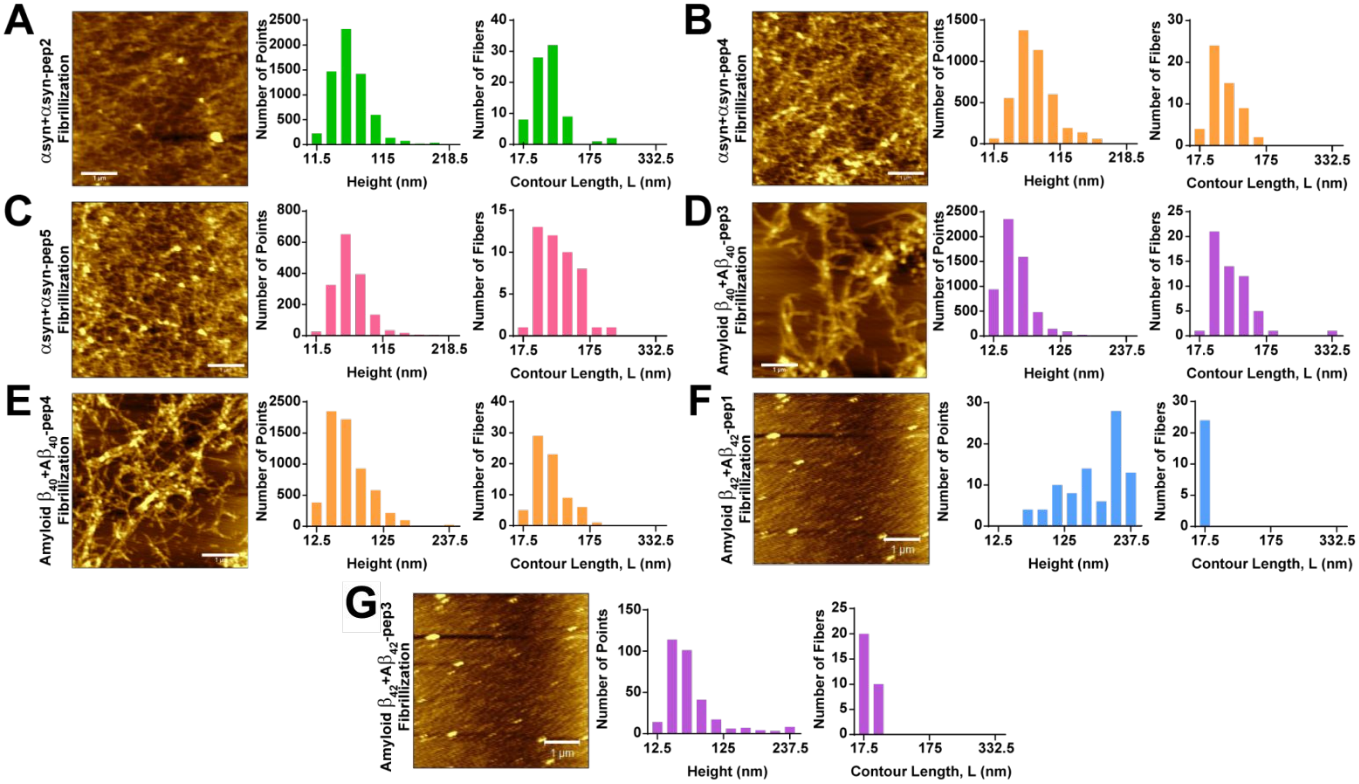

Supplementary Figure S6. AFM analysis for fibrillization assay was applied to determine the effect of peptides in fibrillization. All fibril heights and lengths were analyzed by FiberApp. (A) AFM analysis of α-synuclein+αsyn -pep2 fibrillization product gave a result of web-like aggregation with fibrils. The intensity of α-synuclein+αsyn -pep2 fibrils were not high as the intensity of α-synuclein fibrils, but still, the mesh-like fibril structures were observed with a higher amount of fibril. (B) α-synuclein+αsyn -pep4 fibrillization product AFM analysis gave a result of more intense web-like aggregation with fibrils. When it is compared with the analysis of α-synuclein fibrils, there were more and high amounts of fibrils observed. (C) α-synuclein+αsyn -pep5 fibrillization product AFM result gave a result of more web-like aggregation with fibrils with relatively large contour lengths. Also, the intensity of these fibrils was higher than the intensity of α-synuclein fibrils. (D) AFM analysis of Amyloid β_{40}+Aβ_{40}-pep3 fibrillization product gave a result of web-like aggregation with distinct fibrils. The intensity of the fibrils was less than the only Amyloid β_{40} fibrils. However, some fibrils were observed in higher contour length than the length of only Amyloid β_{40} fibrils. (E) Amyloid β_{40}+Aβ_{40}-pep4 fibrillization AFM analysis gave a result of more intense web-like aggregation with shorter distinct fibrils. The height of the fibrils was relatively shorter than the heights of only Amyloid β_{40} fibrils, although the fibrillization was observed clearly. (F) AFM analysis of Amyloid β_{42}+Aβ_{42}-pep1 fibrillization product gave a result of less amount of seeding with high intensity. These seeds were relatively short than the seeds observed in Amyloid β_{42} fibrillization products. (G) Amyloid β_{42}+Aβ_{42}-pep3 fibrillization AFM analysis gave a result of seed structures other than fibrils. These seeds were less intense, with relatively long contour lengths. Still, there were no high seeding structures observed in Amyloid β_{42} fibrillization products.

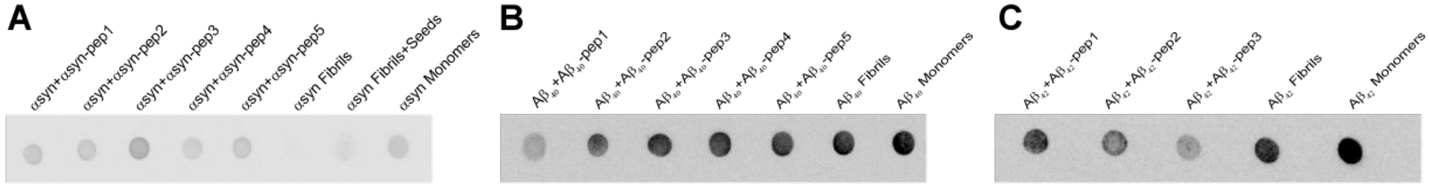

Supplementary Figure S7. Dot-blot analysis was conducted for the detection of fibrillization increase by using monomer-specific antibodies. In the assay, a decrease in monomeric units of NDPs indicates an increase in fibrillization status or effect of peptides to dot-blot assay. (A) Fibrillization status of α-synuclein with and without fibrils was detected. 5 μL of 10 μM α-synuclein samples were analyzed through monomeric unit concentration change. α-synuclein monomers gave more intense dot-spot in the membrane. (B) Monomeric Amyloid β_{40} units were detected for determination of fibrillization status. Although Amyloid β_{40} monomers and Amyloid β_{40}+Aβ_{40}-pep1 fibrillization products gave higher and lower intense dot-spot, respectively, intensity differences of others were less for fibrillization status detection. (C) Monomeric Amyloid β_{42} units were detected for analyzing fibrillization status. There were intensity differences between each dot-spot. Still, there were fewer differences in monomeric Amyloid β_{42} and Amyloid β_{42} fibrils.

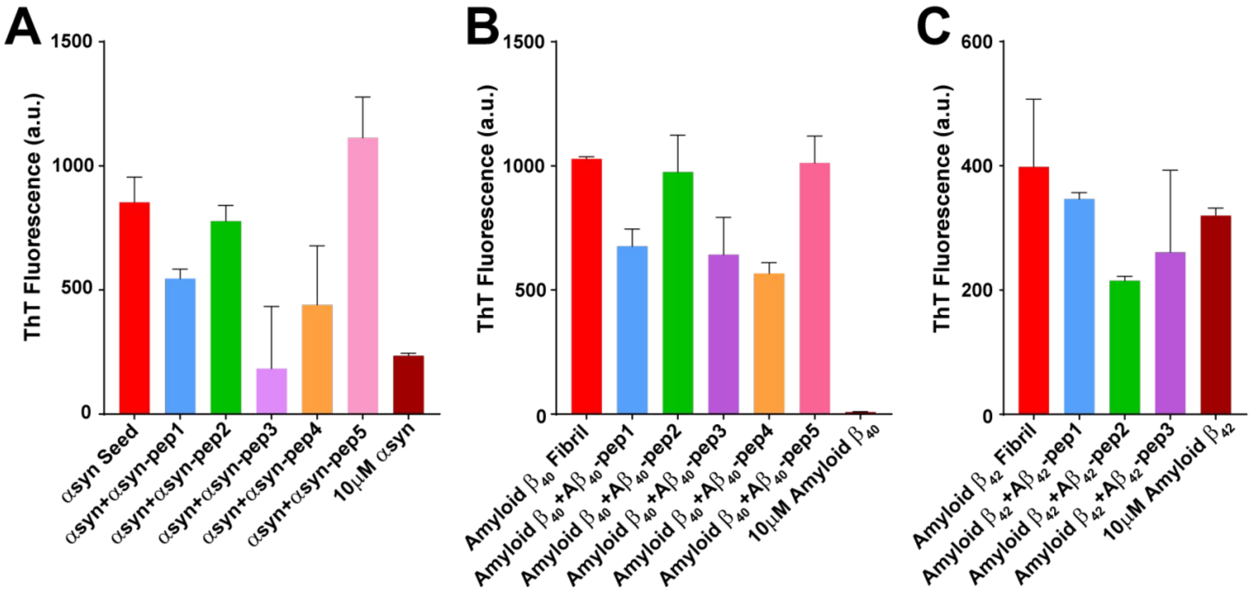

Supplementary Figure S8. End point ThT fluorescence measurements gave different fluorescence signals for each protein-peptide interaction that were used to analyzed by AFM. With the comparison with QCM and AFM analyses, ThT assay also showed the interaction of ThT when NDP interacted with peptides. (A) ThT fluorescence signals were measured for each fibrillization case of α-synuclein. (B) ThT fluorescence signals were measured for each fibrillization case of amyloid β_{40}. (C) ThT fluorescence signals were measured for each fibrillization case of amyloid β_{42}.

Supplementary Methods

Deprotection of resin and coupling with a Fmoc protected amino acid and cleavage reaction during solid phase peptide synthesis. Rink Amide Resin (151.1 mg, 0.05 mmol) (Substitution: 0.331 meq/g) was weighed in a peptide synthesis reactor and it was washed with N, N-dimethylformamide (DMF), and the resin was swollen 20-30 min in 10 mL DMF. After the swelling part, 5 mL of 20% piperidine was poured into the reactor. After 3 min, this part was repeated a second time but 10 min. Then it was washed with DMF. After washing part, Fmoc protected amino acid (5.5 eq., 0.275 mmol) and HBTU (5.0 eq., 0.250 mmol) were weighed in a test tube, and 2.0 mL 0.3 M diisopropylethylamine (DIEA) in DMF was added. After the addition of the DIEA solution, the resulting mixture was added to the reaction vessel and the coupling mixture was left for 1 hour. After the completion of coupling, the resin was washed with DMF extensively. This process was repeated until the desired peptide elongation was obtained. At the end of peptide elongation and the last Fmoc deprotection, the resin was washed with DMF and DCM respectively and left for drying with the pump open for 30 min. After resin was dried, 5 mL cleavage cocktail, 95% TFA (4.75 mL), 5% Milli Q (0.125 mL) and 5% TIPS (0.125 mL) was prepared and added to the reactor. It was incubated for 2 hours and the cleaved peptide solution was precipitated with ice-cold diethyl ether, the solution was centrifuged and the peptide was washed three times with cold ether to remove small organic impurities.

Thioflavin (ThT) Assay. After fibrillization assay, 100 uM α-synuclein and 10 uM peptide, 100 uM uM amyloid β_{40} and 250 uM peptide, 100 uM amyloid β_{42} and 250 uM peptide fibrillization products were added to 96-well plate with 1:10 dilution ratio in 1X PBS. Then NaN_3 and 1M ThT in ddH_2O were added onto each sample to final concentration as 0.1% and 25 uM, respectively. Samples were incubated at 37°C for 3 hours in dark. At the end of the incubation, fluorescence was measured at 450 nm excitation and 485 nm emission with 475 nm cutoff value by SpectraMax M5 microplate reader.

Supplementary References

[1] Benchling [Biology Software]. (2023). Retrieved from https://benchling.com.
[2] Usov, I.; Mezzenga, R. FiberApp: An Open-Source Software for Tracking and Analyzing Polymers, Filaments, Biomacromolecules, and Fibrous Objects. *Macromolecules* **2015**, *48* (5), 1269–1280. https://doi.org/10.1021/ma502264c.
